# Supplementary material for: CEP55 is a determinant of cell fate during perturbed mitosis in breast cancer
Source: EMBO Mol Med. 2018 Aug 14;10(9):e8566. doi: 10.15252/emmm.201708566 (PMC6127888; doi:10.15252/emmm.201708566)

Appendix

CEP55 is a determinant of cell fate during perturbed mitosis in breast cancer

Murugan Kalimutho,[1,2,15*] Debottam Sinha,[1,2,15] Jessie Jeffery,[1] Katia Nones,[1,3] Sriganesh Srihari,[4] Winnie C. Fernando,[1] Pascal H.G. Duijf,[5] Claire Vennin,[6,7] Prahlad Raninga,[1] Devathri Nanayakkara,[1] Deepak Mittal,[1] Jodi M. Saunus,[1,8] Sunil R. Lakhani,[8,9,10] J. Alejandro López,[1,2] Kevin J. Spring,[11,12,13] Paul Timpson,[6,7] Brian Gabrielli,[5,14] Nicola Waddell,[1] Kum Kum Khanna[1*]

[1]QIMR Berghofer Medical Research Institute, 300 Herston Road, Herston, QLD 4006, Australia
[2]School of Natural Sciences, Griffith University, Nathan, QLD 4111, Australia
[3]Queensland Centre for Medical Genomics, Institute for Molecular Bioscience, The University of Queensland, St Lucia, QLD 4072, Australia
[4]Computational Systems Biology Laboratory, Institute for Molecular Bioscience, The University of Queensland, St. Lucia, QLD 4072, Australia
[5]University of Queensland Diamantina Institute, The University of Queensland, Translational Research Institute, Brisbane, QLD 4102, Australia
[6]Garvan Institute of Medical Research and The Kinghorn Cancer Centre, Cancer Division, Sydney, NSW 2010, Australia
[7]St Vincent's Clinical School, Faculty of Medicine, University of NSW, Sydney, NSW 2010, Australia.
[8]The University of Queensland, Centre for Clinical Research, Herston, QLD 4029, Australia
[9]The University of Queensland, School of Medicine, Herston 4029, Queensland, Australia
[10]Pathology Queensland, The Royal Brisbane and Women's Hospital, Herston, QLD 4029, Australia
[11]Liverpool Clinical School, University of Western Sydney, Locked bag 7103, Liverpool 1871 Australia
[12]Ingham Institute, Liverpool Hospital Liverpool, NSW 2170, Australia
[13]South Western Sydney Clinical School, University of New South Wales, Liverpool, NSW 1871
[14]Mater Research Institute, The University of Queensland, Translational Research Institute, Brisbane, Queensland, Australia
[15] These authors contributed equally
***Correspondence:** Murugan.Kalimutho@qimrberghofer.edu.au (M.K)
KumKum.Khanna@qimrberghofer.edu.au (K.K.K)

Table of Contents

Appendix Figure Legends
Appendix Tables S1-5
Appendix Figures S1-9

Appendix Fig S1:

(A) Overall survival, relapse-free survival and distant metastasis-free survival of clinical analysis as describe in Figure EV D-F using the KMplotter online tool (http://kmplot.com/)(Gyorffy et al, 2010). *CEP55* expression using the breast cancer TCGA dataset after normalization to Ki67 **(B)**, or PCNA **(C),** (expressed as ratio) and their association with subtypes (right panels).

Appendix Fig S2: (A) Subtype-specific *CEP55* mRNA expression (Log2 expression) in breast cancer lines assessed using GOBO software. Neve dataset was used to derive this plot (Neve et al, 2006). Graph was obtained from GOBO online tool. **(B, C)** Interim analyses of *CEP55* mRNA expression (Log2 expression) in basal-like *vs.* non-basal-like breast cancer cell lines (TNBC: triple-negative breast cancer; HR pos: Hormone receptor positive) as described in panel A.

(D) Left, Immunoblot analysis showing doxycycline-inducible (2µg/ml) knockdown of CEP55 in MDA-MB-231 cells 48-hour post induction. Isogenic lines were established using two different CEP55-specific shRNAs (sh#2 & sh#8) and scramble shRNA as a control, see method for sequence details. COX-IV as a loading control. Right, Densitometry analysis of both baseline and doxycycline-induced CEP55 reduction was quantitated using Image J software. Graph represents the mean±SEM of three independent experiments.

Appendix Fig S3:

(A, B) Cell migration and invasion index (rate) was determined using the XCELLigence system (Dunne et al, 2014). For the migration assay, serum-free media was used in top the chambers and 10% serum contained media was used in the bottom chambers and the migration rate was determined in real time. For the invasion assay, top chambers were coated

with 100% BD growth factor reduced Matrigel and bottom chambers contained 10% serum contained media. 0.1 million cells were seeded for each analysis. Representative images are shown for cell migration and invasion (bottom panel). Graphs represent the mean±SEM of two independent experiments.

(C) Representative images of excised tumors of control and CEP55 knockdown (sh#2) MDA-MB-231 xenograft. Data for these images are shown in Figure 1G.

(D) Effect of CEP55 overexpression on cell migration in MCF10A lines assessed using the xCELLigence cell tracking system as described in panel A. Graph represents the mean±SEM of two independent experiments.

(E) Quantification of crystal violet intensity (absorbance value at 540 nM) for Figure 1J.

Appendix Fig S4:

(A) Percentage of breast cancer TCGA tumors with and without chromosome 20q gain and loss, $P<0.0001$, Chi-square test.

(B) *CEP55* expression in TCGA tumors that were stratified with and without chromosome 20q gain.

Appendix Fig S5:

(A) Average time spent in mitosis of growing both control and CEP55 knockdown MDA-MB-231 cells. Time taken to complete mitosis was defined as the time from nuclear envelope breakdown until two daughter cells were observed. For each experiments n=50 mitotic cells were counted per condition using Olympus Xcellence IX81 time-lapsed microscopy. Graph represents the mean±SEM of two independent experiments.

Representative images of mitotic slippage in control **(B)** and mitotic cell death in sh#2 and sh#8 MDA-MB-231 cells are shown **(C, D)**.

Appendix Fig S6:

(A) Control and CEP55 knockdown MDA-MB-231 cells were synchronized by double-thymidine block and released into culture medium. Cells were then collected every 2 hour interval for cell cycle profiling. Graph represents the mean±SEM of two independent experiments.

(B, C) Similar to experiment in panel A, synchronized control and CEP55 knockdown MDA-MB-231 cells were released into either B12536 (5 nM) or nocodazole (0.5 µM) and phases cell cycle distribution and **(D, E)** subG1 population were determined. Graph represents the mean±SEM of two independent experiments.

(F, J) Average time to mitosis **(G, K)** Average time spent in mitosis and **(H, L)** mitotic outcomes in control and CEP55 knockdown MDA-MB-231 or CEP55-overespressing MCF10A cells following treatment with nocodazole (0.5 µM). Graph represents the mean±SEM of two independent experiments. For each experiments n=50 mitotic cells were counted per condition using Olympus Xcellence IX81 time-lapse microscopy.

(I) Both control and CEP55 knockdown Hs578T lines were synchronized using double thymidine then released into nocodazole (0.5 µM), and protein lysates were collected at the indicated time points. Immunoblot analysis was then performed to determine the expression and activity of mitotic regulators as indicated. Levels of phospho-MEK^{T286} and dephosphorylation of phospho-CDK1^{Y15} served as markers of Cdk1 activation/mitotic entry. COX-IV served as a loading control.

(M) Cells were synchronized as above, and released into 0.25 µM nocodazole for 24 h for immunoblot analysis of the indicated mitotic markers.

Appendix Fig S7:

(A) Relative fold changed of *CEP55* and *MYC* mRNA levels following different MEK1/2 inhibitors treatment at indicated doses and time. Fold changed was calculated relative to untreated control cells. Graphs represent the mean±SEM of two independent experiments.

(B) Immunoblot showing impact of AZD6244 (0.5 µM) treatment on *CEP55* and *MYC* levels in MDA-MB-231 cells at indicated time points. COX-IV as a loading control.

(C) Quantitation of cell cycle distribution of MDA-MB-231 cells treated with different MEK1/2 inhibitors (selumetinib (1 µM) or Trametinib (0.5 µM)) for indicated time points. Graph represents the mean±SEM of two independent experiments.

(D) Relative *CEP55* promoter luciferase activity upon 10 nM *ERK1/2* siRNA determined using DualGlo assay in MDA-MB-231cells similar to experiment in Figure 4E. PGL basic vector was used to normalize *CEP55*-promoter activity. Graph represents the mean±SEM of two independent experiments.

(E) Relative fold changed of *CEP55* and *MYC* mRNA levels following EGF stimulation in MDA-MB-231 cells cultured in 0.1% fetal bovine serum at indicated time points. Relative fold changed was calculated to untreated control cells. Graph represents the mean±SEM of two independent experiments.

(F) Relative basal and EGF induced fold changed of *CEP55, MYC* and *ETS1* mRNA levels at indicated time points in MDA-MB-231 cells transfected with siRNA against 10 nM *CEP55, MYC* or *ETS1* for 24 hour. Graph represents the mean±SEM of two independent experiments.

Appendix Fig S8:

(A) Immunoblots showing CEP55 expression in control and CEP55 knockdown Hs578T cell lines. COX-IV as a loading control.

(B) Both control and CEP55 knockdown Hs578T cells were exposed with different concentrations of BI2536 alone (i) or in combination with AZD6244 (1 µM) (ii-iii), and cell viability was determined after 6 days. The dose-response curve was generated by calculating cell viability relative to untreated control and plotted against drug concentration. Graph represents the mean±SEM of three independent experiments.

(C) Percentage of sub-G1 population identified using propidium iodide staining and quantified by FACS following single and combination treatment with AZD6244 and BI2536 inhibitors after 96h in control and CEP55 knockdown Hs578T cells. Graph represents the mean±SEM of two independent experiments.

(D) Immunoblots analysis of both control and CEP55 knockdown Hs578T cells treated with single and combination treatment with AZD6244 (1 µM) and BI2536 (5 nM) inhibitors after 96h. Cleaved PARP, Caspase-3 along with MYC, ERK1/2 and CEP55 were determined. COX-IV as a loading control.

(E) Immunoblots analysis as described in panel D in control, sh#8 and sh#8rescue. The shRNA-resistant construct was transiently transfected with 1 µg of DNA for 48 h followed by indicated treatment in sh#8 cells.

(F) Percentage of sub-G1 analysis as described in panel C in CEP55 overexpressing MCF10A cells. Graph represents the mean±SEM of two independent experiments.

(G,H) Immunoblots analysis was performed in a panel of breast cancer cell lines treated with single or in combination with AZD6244 and BI2536 inhibitors after 96 hours. Cleaved PARP and Caspase-3 were determined along with CEP55, FOXM1, MYC, phosphorylated and total ERK1/2. COX-IV as a loading control (left panels). Percentage of sub-G1 population identified using propidium iodide staining and quantified by FACS following single and combination treatment with AZD6244 and BI2536 inhibitors after 96h (middle panels). Graph represents the mean±SEM of two independent experiments. Representative images of

colony forming capacity at 14 days determined using crystal violet staining in cells treated with single and combination inhibitors (middle panels).

Appendix Fig S9:

(A) Growth rate (mean tumor size, area, mm^2) of pre-treated six week old female BALB/c cohorts of mice bearing the 4T1.2 mammary tumor line, n=6 mice per group.

(B) Left, Growth rate (mean tumor size, area, mm^2) of MDA-MB-231-HM_LNm5 xenografts in six week old BALB/c Nude mice treated with vehicle, AZD6244, BI6727, or combined AZD6244/BI6727 treatment as indicated in Figure 6D, n=6 mice/group. Right, representative excised tumors are shown.

References

Chou TC, Talalay P (1984) Quantitative analysis of dose-effect relationships: the combined effects of multiple drugs or enzyme inhibitors. Adv Enzyme Regul 22**:** 27-55

Dunne PD, McArt DG, Blayney JK, Kalimutho M, Greer S, Wang T, Srivastava S, Ong CW, Arthur K, Loughrey M et al (2014) AXL is a key regulator of inherent and chemotherapy-induced invasion and predicts a poor clinical outcome in early-stage colon cancer. Clin Cancer Res 20**:** 164-175

Gyorffy B, Lanczky A, Eklund AC, Denkert C, Budczies J, Li Q, Szallasi Z (2010) An online survival analysis tool to rapidly assess the effect of 22,277 genes on breast cancer prognosis using microarray data of 1,809 patients. Breast cancer research and treatment 123**:** 725-731

Neve RM, Chin K, Fridlyand J, Yeh J, Baehner FL, Fevr T, Clark L, Bayani N, Coppe JP, Tong F et al (2006) A collection of breast cancer cell lines for the study of functionally distinct cancer subtypes. Cancer cell 10**:** 515-527

Cell Lines / BI2536 (nM)	Combination Index (CI)					
	0.5	1.0	2.5	5.0	10.0	AZD6244 (µM)
**MDA-MB-231**	0.63811	0.60042	0.31312	0.21833		1.0
	0.74101	0.37383	0.26706	0.18501		2.5
	0.37502	0.26472	0.21285	0.06295		5.0
**MDA-MB-231-HM**	1.01792	0.98665	0.66793	0.16865		1.0
	0.7554	0.6076	0.5003	0.2748		2.5
	0.67342	0.56043	0.35688	0.30862		5.0
**Hs578T**	0.4206	0.52828	0.80064	0.16488		1.0
	0.832028	0.37736	0.52481	0.66563		2.5
	0.78932	0.65043	0.7532	0.2075		5.0
**MDA-MB-468**	0.53875	0.70595	0.76962	0.17903		1.0
	0.64273	0.55106	0.5839	0.26456		2.5
	1.16994	0.87957	0.78996	0.40029		5.0
**MDA-MB-436**	0.5506	0.52286	0.35436	0.10167		1.0
	0.45531	0.21339	0.26332	0.06264		2.5
	0.39947	0.21463	0.11989	0.11761		5.0
**MDA-MB-157**	0.59691	0.64342	0.42106	0.38059		1.0
	0.51258	1.32769	0.42771	0.24732		2.5
	0.41175	0.2491	0.20778	0.12427		5.0
**SUM159PT**		22.7	20.29	24.76	0.706	1.0
		17.7521	31.958	36.6107	0.6804	2.5
		1.40111	1.5238	1.64153	0.29184	5.0
**BT549**	6.3373	1.86307	0.51791	0.78561		1.0

	12.1611	7.65173	0.86976	0.73594		2.5
	41.661	21.1836	0.74087	0.74513		5.0
**SKBR3**	7.71315	1.94906	2.14818	55.4718		1.0
	7.67319	23.5828	5.49653	22.1958		2.5
	2.9599	2.22675	3.06897	7.12916		5.0
**MCF7**	7.71315	1.94906	2.14818	55.4718		1.0
	7.67319	23.5828	5.49653	22.1958		2.5
	2.9599	2.22675	3.06897	7.12916		5.0

Appendix Table S1: Combination index (CI)(Chou & Talalay, 1984) following combined AZD6244-BI2536 treatment in a panel of breast cancer cell lines.

Gene Name	Sequence	
	sense（5'-3'）	antisense（5'-3'）
c-MYC_5	AUGUAAACUGCCUCAAAUUGGACTT	AAGUCCAAUUUGAGGCAGUUUACAUUA
c-MYC_CDS	GCGACGAGGAGGAGAACUUCUACCA	UGGUAGAAGUUCUCCUCCUCGUCGCAG
ETS-1_5	CCCAGAGAUGCCUUAACCUUUGUTG	CAACAAAGGUUAAGGCAUCUCUGGGAA
ETS-1_1	CCAGAAGAGAGGAAUGACUUGAAGG	CCUUCAAGUCAUUCCUCUCUUCUGGAA
ERK2/MAPK1_1	CCAGGAUACAGAUCUUAAAUUUGTC	GACAAAUUUAAGAUCUGUAUCCUGGCU
ERK1/MAPK3	AUAAACGGAUCACAGUGGAGGAAGC	GCUUCCUCCACUGUGAUCCGUUUAUUG
CEP55_1	GUCCCAAGUGCAAUAUACAGUAUCC	GGAUACUGUAUAUUGCACUUGGGACAU
CEP55t2_2	GCAACAUCUGGAAGAUGAUAGGCAT	AUGCCUAUCAUCUUCCAGAUGUUGCAC
CEP55_3	CCCUGACAUGGUUCAUCAUCAGGCT	AGCCUGAUGAUGAACCAUGUCAGGGAG

Appendix Table S2: siRNAs used in this study

Gene Name	Sequence	
	Forward	Reverse
ETS1	TCATTTCTTTGCTGCTTGGA	CTCACCATCATCAAGACGGA
CEP55	TGGCTCCAAACTGCTTCAAC	ACTTCCCGCTGCTGATCATA
MYC	ACCGAGTCGTAGTCGAGGT	TTTCGGGTAGTGGAAACCA
ATCB	CCCAGAGCAAGAGAGAGG	GTCCAGACGCAGGATG
HPRT1	CCTGGCGTCGTGATTAGTGAT	AGACGTTCAGTCCTGTCCATAA

Appendix Table S3: PCR primers used in this study

**Antibody Name**	**Company**	**Cat. No**	**Dilution**
CEP55	In-house (RB1)	-	1:4000
γ-Tubulin	Sigma Aldrich	T5192	1:1000
β-Actin	BD Pharmingen	612656	1:2000
COX-IV	Millenium Science Pty Ltd	LCR-926-42214	1:2000
P53	Santa Cruz	Sc-126	1:1000
β-Catenin	Cell Signaling Technology	9582	1:1000
ZEB/TCF	Cell Signaling Technology	3396	1:1000
Vimentin	Cell Signaling Technology	5741	1:1000
pSTAT3(Y705)	Cell Signaling Technology	9145	1:1000
STAT3	Cell Signaling Technology	9139	1:1000
pAKT (S473)	Cell Signaling Technology	4060	1:1000
AKT	Cell Signaling Technology	9272	1:1000
pERK1/2(T202/Y204)	Cell Signaling Technology	4370	1:2000
ERK1/2	Cell Signaling Technology	4695	1:2000
pEGFR (Y1068)	Cell Signaling Technology	2234	1:1000
PARP	Cell Signaling Technology	9542	1:1000
Cleaved Caspase-3	Cell Signaling Technology	9664	1:500
MYC (Y69)	Abcam	Ab32072	1:1000
AURKA	Cell Signaling Technology	4178	1:1000
MPM2	Upstate biotechnology	05-368	1:500
Cyclin B1	Abcam	Ab7957	1:1000
p-MEK(T286)	Cell Signaling Technology	9127	1:1000
p-CDK1(Y15)	Cell Signaling Technology	4539	1:1000
WEE1	Cell Signaling Technology	4936	1:1000
CDC25B	Sigma Aldrich	Sc-5619	1:250
p-MCL1(S159/T163)	Cell Signaling Technology	4579	1:1000
p-H3 (S10)	Cell Signaling Technology	9706	1:1000
BCL2	Cell Signaling Technology	2876	1:1000
BCL-XL	BD Pharmingen	51-9000093	1:1000
BAK	Pro Sci Incorporated	3347	1:1000
BIM	Cell Signaling Technology	2933p	1:1000
Cytokeratin 19 for IF	Abcam	ab15463	1:10
Survivin	GeneTex Inc	GTX100441	1:1000

Appendix Table S4: List of antibodies used in this study.

**Figure**	**Statistical significant (p)**	**Test used**
Fig 1E	< 0.0001	2
Fig 1G	< 0.0001	3
Fig 1I	EV *vs.* #16 : 0.0142<br>EV *vs.* #16 : 0.0263	3
Fig 2B	T test: <0.0001<br>F test: <0.0001	1
Fig 2D	shSCR *vs.* sh#2 or sh#8:< 0.0001	2
Fig 2F	< 0.0001	2
Fig 2G	< 0.0001	4
Fig 3B	shScr (DMSO *vs.* BI2536): <0.0001<br>sh#2 (DMSO *vs.* BI2536): 0.0003<br>sh#8 (DMSO *vs.* BI2536): 0.0697<br>ShScr BI2536 *vs.* #2 and #8 BI2536: <0.0001	1, 3
Fig 3C	shScr (DMSO *vs.* Nocodazole): 0.0007<br>shScr Nocodaxole *vs.* sh#2 and sh#8 Nocodazole: <0.0001	1, 3
Fig 3D	shScr (DMSO *vs.* BI2536): 0.0043<br>sh#2 (DMSO *vs.* BI2536): <0.0001<br>sh#8 (DMSO *vs.* BI2536): <0.0001<br>shScr BI2536 *vs.* sh#2 and sh #8 BI2536: 0.0013	1,3
Fig 3E	shScr (DMSO *vs.* nocodazole): <0.0001<br>sh#2 (DMSO *vs.* nocodazole): <0.0001<br>sh#8 (DMSO *vs.* nocodazole): <0.0001<br>shScr nocodazole *vs.* sh#2 nocodazole:<0.001<br>shScr nocodazole *vs.* sh#8 nocodazole: <0.0001	1,3
Fig 3F	shScr DMSO *vs.* nocodazole: 0.0019<br>shCEP55 DMSO *vs.* BI2536: 0.0210<br>shCEP55 DMSO *vs.* nocodazole:0.0007<br>shSCR mitotic-inhibitors *vs.* shCEP55 mitotic inhibitors: 0.0446	1,3
Fig 3H	EV (DMSO *vs.* BI2536): 0.0427<br>C17 (DMSO *vs.* BI2536): < 0.0001<br>C18 (DMSO *vs.* BI2536): < 0.0001<br>EV BI2536 *vs.* C17 and C18 BI2536: < 0.0001	1,2
Fig 3I	shScr (DMSO *vs.* BI2536): 0.0075<br>sh#2 (DMSO *vs.* BI2536): 0.0012<br>sh#8 (DMSO *vs.* BI2536): 0.0008<br>shScr BI2536 *vs.* sh#2 and sh #8 BI2536: 0.0263	1,2
Fig 4A	<0.0001	2
Fig 4B	<0.0001	2
Fig 4C	<0.0001	3
Fig 4D	<0.0001	2
Fig 4H	8h: shSCR *vs.* shCEP55: <0.0001<br>10h: shSCR *vs.* shCEP55: 0.0069	2
Fig 4I	<0.0001	2,3
Fig 4J	<0.0001	2
Fig 5C	P1 *vs.* MEK1/2i :0.0127	1
Fig 5E	Basic *vs.* P1: 0.0332	1

	P1 *vs.* siMYC: 0.0309	
Fig 5H	0.0016	5
Fig 5I	<0.0001	5
Fig 5K	shSCR: Combo *vs.* MEK1/2i or PLK1i: <0.0001<br>sh#2:Combo *vs.* PLK1i:0.0430<br>sh#8:Combo *vs.* PLK1i: <0.0001	2
Fig 6B	<0.0001	2
Fig 6C	<0.0001	3
Fig 7A	Vehicle *vs.* MEK1/2i 12.5mg/kg BID:0.0919<br>Vehicle *vs.* PLK1i 12.5mg/kg: 0.2254<br>Vehicle *vs.* Combination at day 10: <0.0001	2
Fig 7C	0.0005	4
Fig 7D	Vehicle *vs.* MEK1/2i 12.5mg/kg BID:0.0035<br>Vehicle *vs.* PLK1i 12.5mg/kg:<0.0001<br>Vehicle *vs.* Combination:<0.0001	2
Fig 7E	<0.0001	5
Fig EV1A-c	<0.0001	6
Fig EV1D	0.00102	6
Fig EV1E	<0.00001	6
Fig EV1F	0.01135	6
Fig EV2B	<u>BT549</u><br>Cell confluency: <0.0001<br>Sub-G1: 0.0010<br><u>MDA-MB-436</u><br>Cell confluency: <0.0001<br>Sub-G1: 0.0164	1
Fig EV2C	shSCR *vs.* sh#8: 0.0184	2
Fig EV2E	p<0.0001	2
Fig EV2F	<0.0001	2
Fig EV3B	<0.0001	3
Fig EV3D	< 0.0001	1
Fig EV3L	0.0150	1
Fig EV4A	< 0.0001	2
Fig EV4B	< 0.0001	2
Fig EV5B,C	< 0.0001	3
Appendix Fig S1B	<0.0001	2
Appendix Fig S1C	<0.0001	2
Appendix Fig S3A	<0.0001	2
Appendix Fig S3B	shSCR *vs.* sh#2: 0.0198<br>shSCR *vs.* sh#8:0.0027	2
Appendix Fig S3D	<0.0001	2
Appendix Fig S3E	<0.0001	2
Appendix Fig S4A	$1.16x10^{-34}$	7

Appendix Fig S4B	<0.0001	1
Appendix Fig S6D	PLK1i<br>4h:shSCR *vs.* 4h:sh#8:0.0105<br>6h:shSCR *vs.* 6h:sh#8:0.0008<br>8h:shSCR *vs.* 8h:sh#8:<0.0001<br>10h:shSCR *vs.* 10h:sh#8:<0.0001<br>12h:shSCR *vs.* 12h:sh#2:0.0004<br>12h:shSCR *vs.* 12h:sh#8:<0.0001	3
Appendix Fig S6E	Nocodazole<br>4h:shSCR *vs.* 4h:sh#8:0.0060<br>6h:shSCR *vs.* 6h:sh#8:0.0187<br>8h:shSCR *vs.* 8h:sh#2:0.0076<br>8h:shSCR *vs.* 8h:sh#8:<0.0001<br>10h:shSCR *vs.* 10h:sh#8:<0.0001<br>12h:shSCR *vs.* 12h:sh#2:<0.0001<br>12h:shSCR *vs.* 12h:sh#8:<0.0001	3
Appendix Fig S6F	shSCR *vs.* sh#8:<0.0001<br>shSCR *vs.* sh#8rescue:0.9137	2
Appendix Fig S6G	shSCR *vs.* sh#8:0.0436	2
Appendix Fig S6J	EV *vs.* C#17: <0.0001	1
Appendix Fig S6K	EV *vs.* C#17: 0.0303	1
Appendix Fig S7A	*CEP55* mRNA<br>24h:DMSO *vs.* Trematinib 0.5uM:0.0221<br>48h:DMSO *vs.* Selumetanib 1.0uM:0.0082<br>48h:DMSO *vs.* Trematinib 0.5uM:0.0041<br>*MYC* mRNA<br>12h:DMSO *vs.* Trematinib 0.5uM:0.0472<br>24h:DMSO *vs.* Trematinib 0.5uM:0.0169<br>48h:DMSO *vs.* Selumetanib 1.0uM:0.0082<br>48h:DMSO *vs.* Trematinib 0.5uM:0.0031	3
Appendix Fig S7D	Basic *vs.* P1: 0.0032<br>P1 *vs.* siERK1/2:0.0052	2
Appendix Fig S7E	*CEP55* mRNA<br>0h *vs.* 60min: 0.0564<br>*MYC* mRNA<br>0h *vs.* 30min: 0.0326<br>0h *vs.* 60min: 0.0106	2
Appendix Fig S8C	shSCR<br>Combo *vs.* PLK1i or MEK1/2i:<0.0001<br>shCEP55<br>Combo *vs.* PLK1i:<0.0001	2
Appendix Fig S8F	EV<br>DMSO *vs.* combo: 0.0251<br>#C17<br>DMSO *vs.* combo: <0.0001<br>Combo EV *vs.* Combo C#17: <0.0001	3
Appendix Fig S8G	<0.0001	2
Appendix Fig S9B	Day 15<br>Vehicle *vs.* MEK1/2i 12.5mg/kg BID: 0.3393	2

	Vehicle *vs.* PLK1i 12.5mg/kg: 0.1107<br>Vehicle *vs.* Combination: <0.0001	

Appendix Table S5: Statistical significance p value for each figure.
1: Pair or unpaid T test; 2: one-way ANOVA with post-hoc Bonferroni; 3: two-ways ANOVA with post-hoc Bonferroni; 4: Log-rank (Mantel-Cox) test; 5: Pearson correlation co-efficient; 6: http://co.bmc.lu.se/gobo/gsa.pl; 7: Chi-square.

Appendix Figure S1

A.

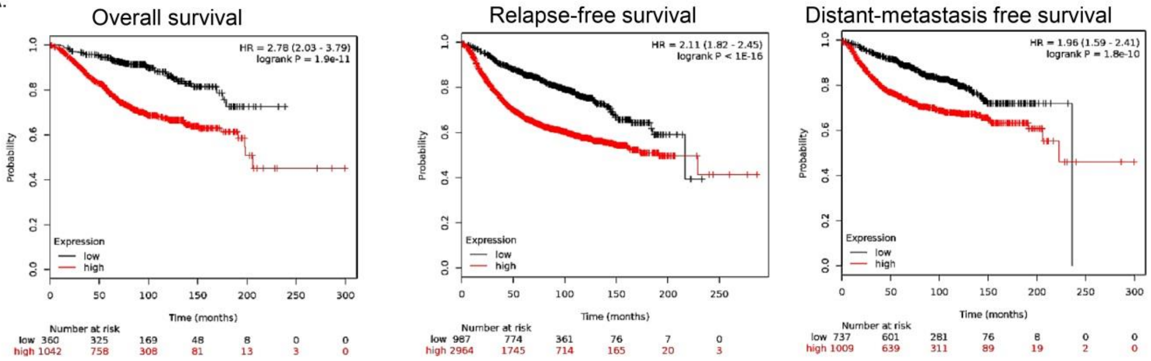

Median survival

Low expression cohort (months)	High expression cohort (months)	Low expression cohort (months)	High expression cohort (months)	Low expression cohort (months)	High expression cohort (months)
179.01	70	216.66	191.21	236.22	222.81

B.

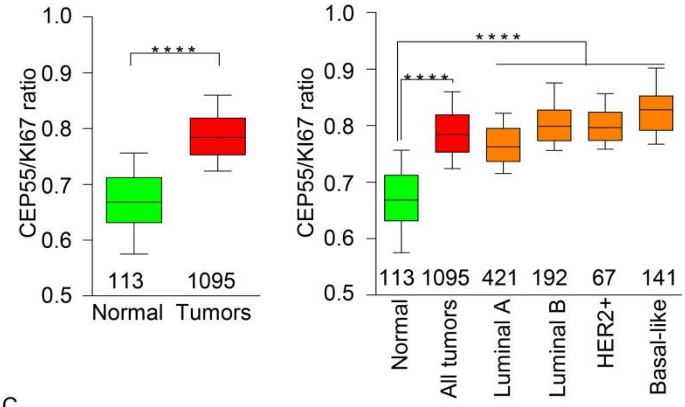

C.

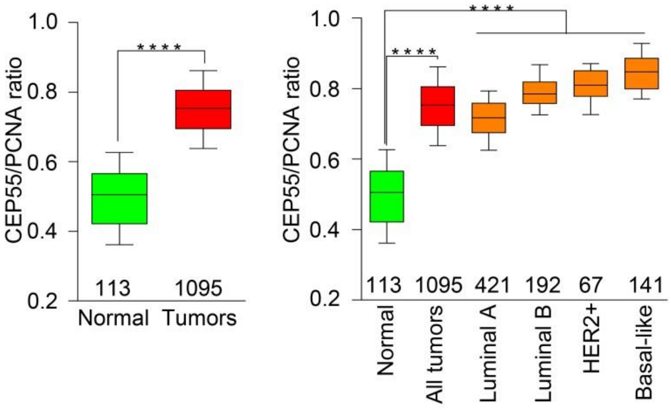

Appendix Figure S2

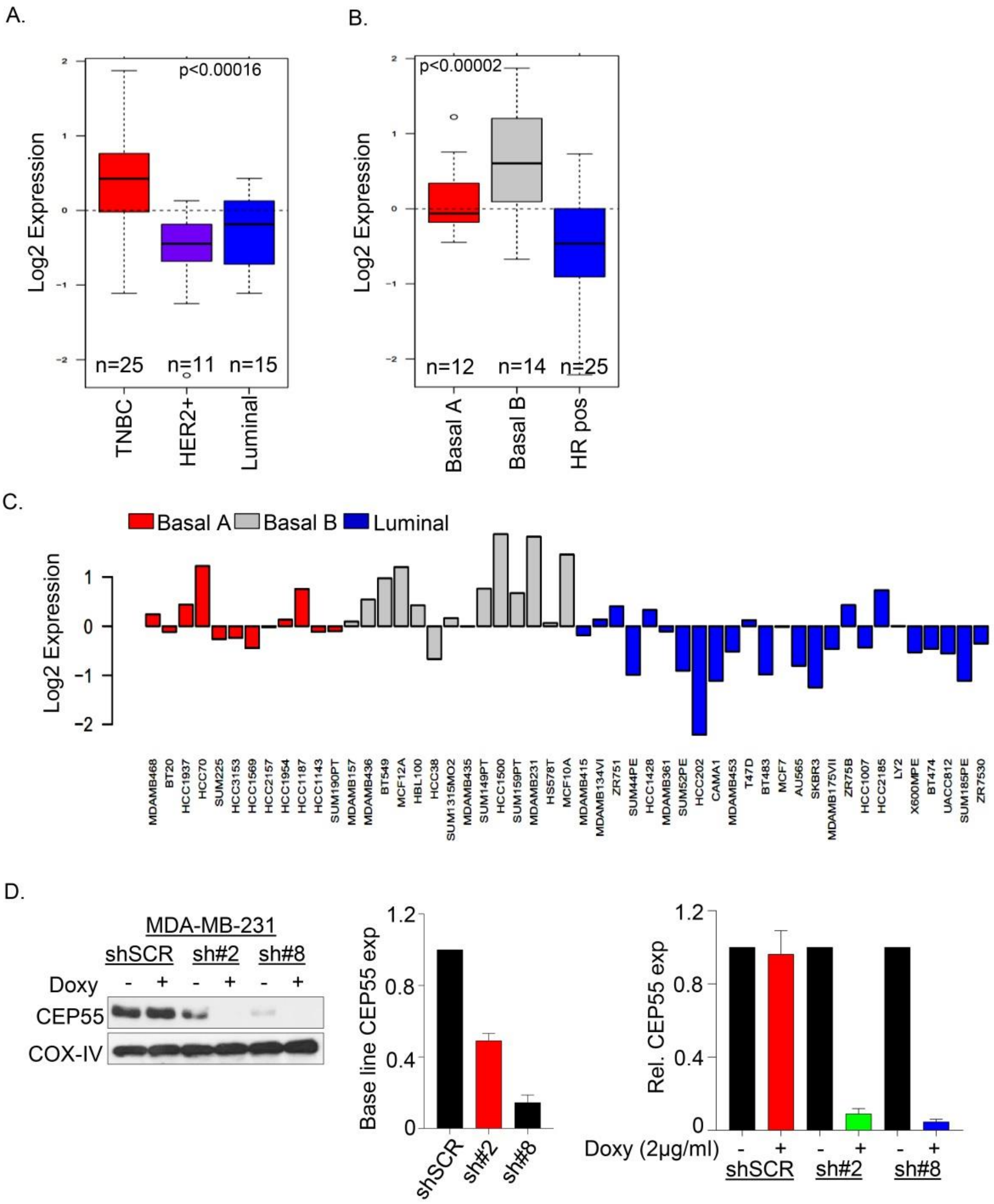

Appendix Figure S3

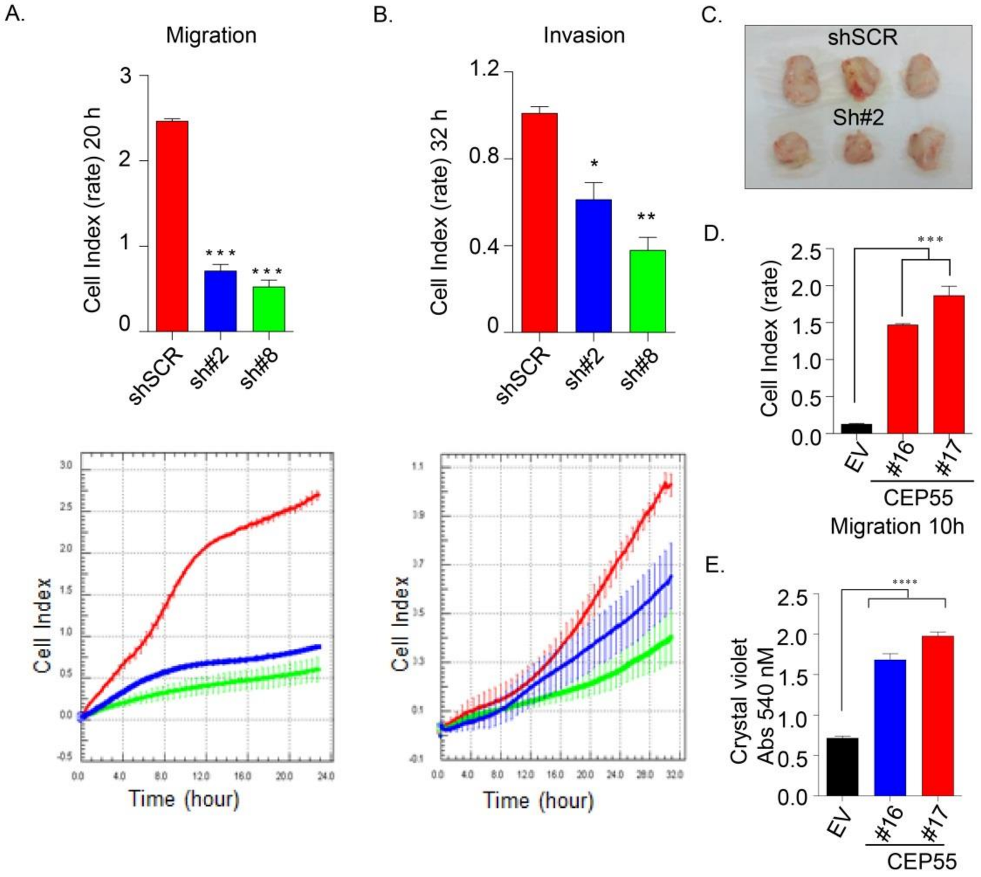

Appendix Figure S4

A.

	+20q	-20q		+20q	+20q
#tumors with	171	12	%	15.6	1.1
#tumors without	923	1082	%	84.4	98.9
Chi-square	$P=1.16x10^{-34}$				

B.

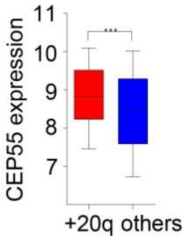

Appendix Figure S5

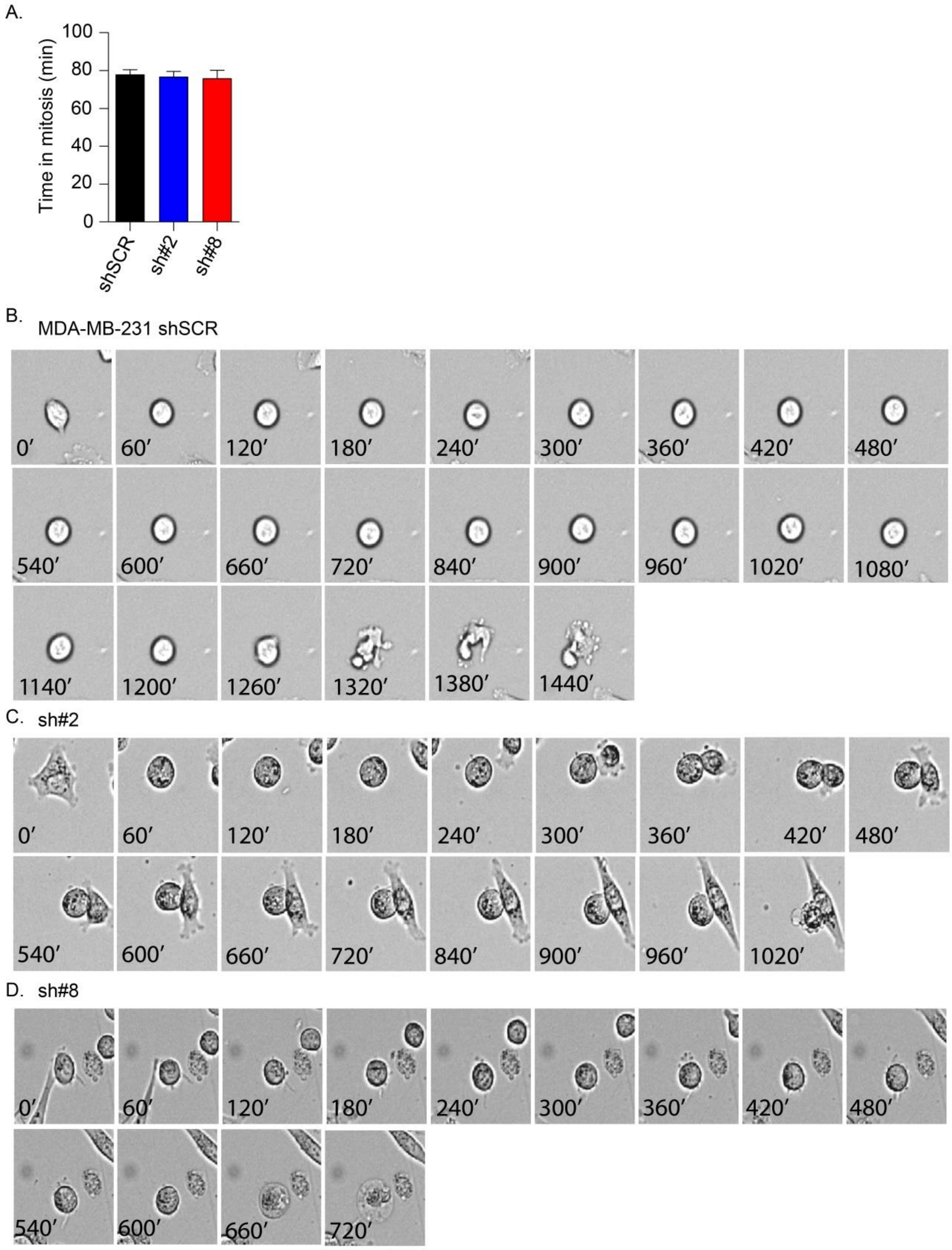

Appendix Figure S6

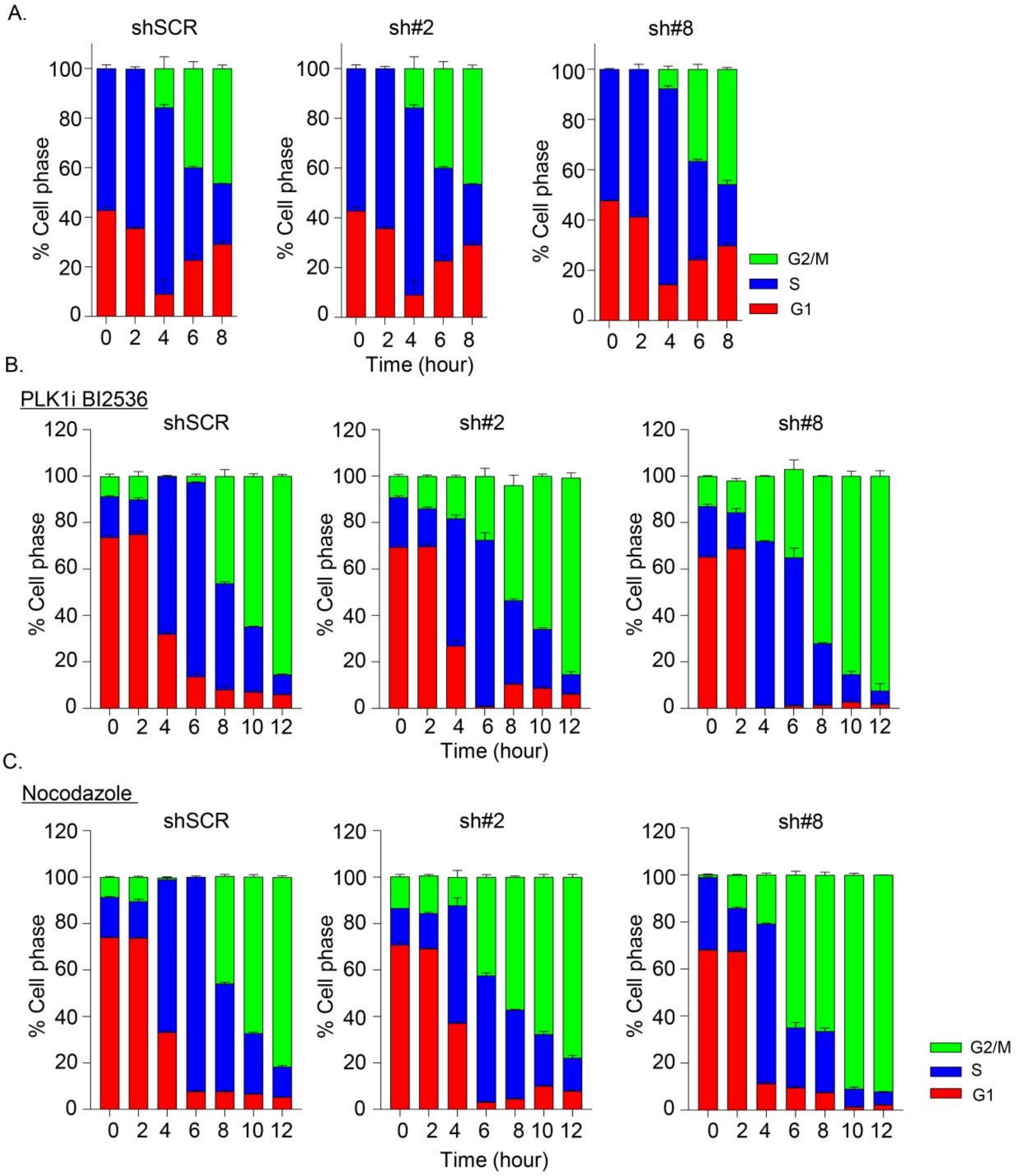

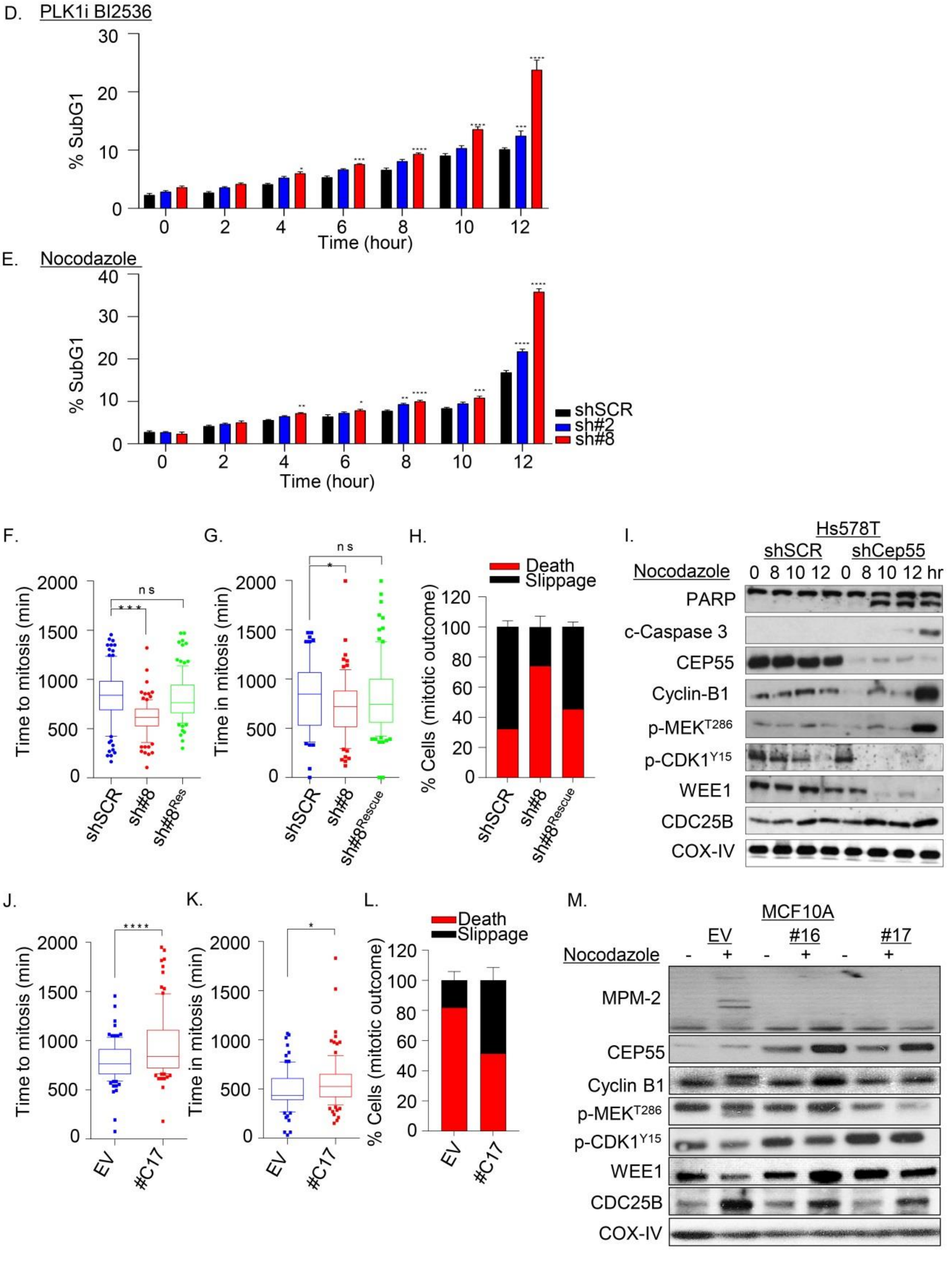

D. PLK1i BI2536
% SubG1
Time (hour)
E. Nocodazole
% SubG1
Time (hour)
shSCR
sh#2
sh#8
F.
Time to mitosis (min)
shSCR
sh#8
sh#8Res
G.
Time in mitosis (min)
shSCR
sh#8
sh#8Rescue
H.
Death
Slippage
% Cells (mitotic outcome)
I.
Hs578T
shSCR
shCep55
Nocodazole
0 8 10 12 0 8 10 12 hr
PARP
c-Caspase 3
CEP55
Cyclin-B1
p-MEKT286
p-CDK1Y15
WEE1
CDC25B
COX-IV
J.
Time to mitosis (min)
EV
#C17
K.
Time in mitosis (min)
L.
Death
Slippage
% Cells (mitotic outcome)
M.
MCF10A
EV
#16
#17
Nocodazole
MPM-2
CEP55
Cyclin B1
p-MEKT286
p-CDK1Y15
WEE1
CDC25B
COX-IV

Appendix Figure S7

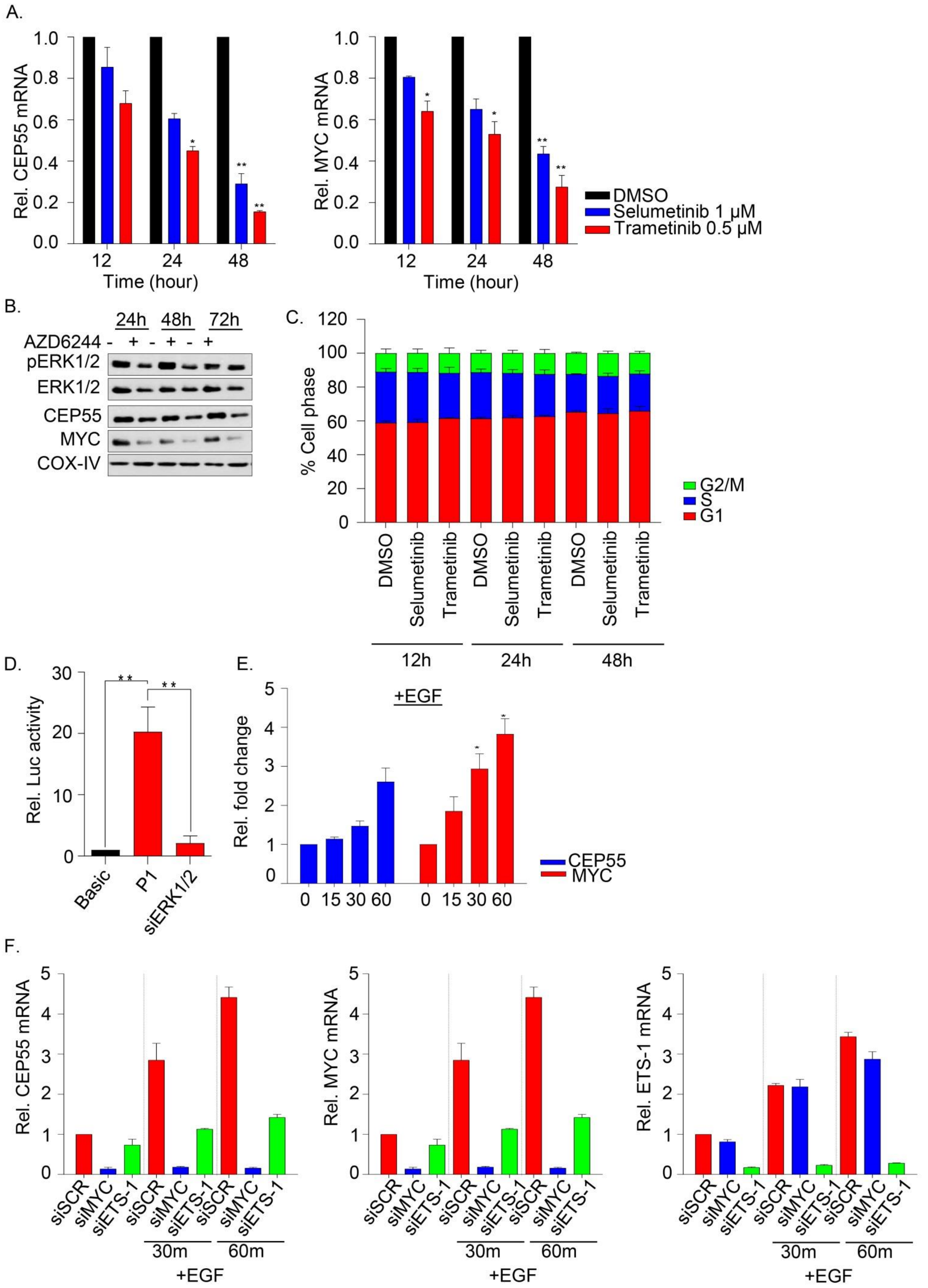

Appendix Figure S8

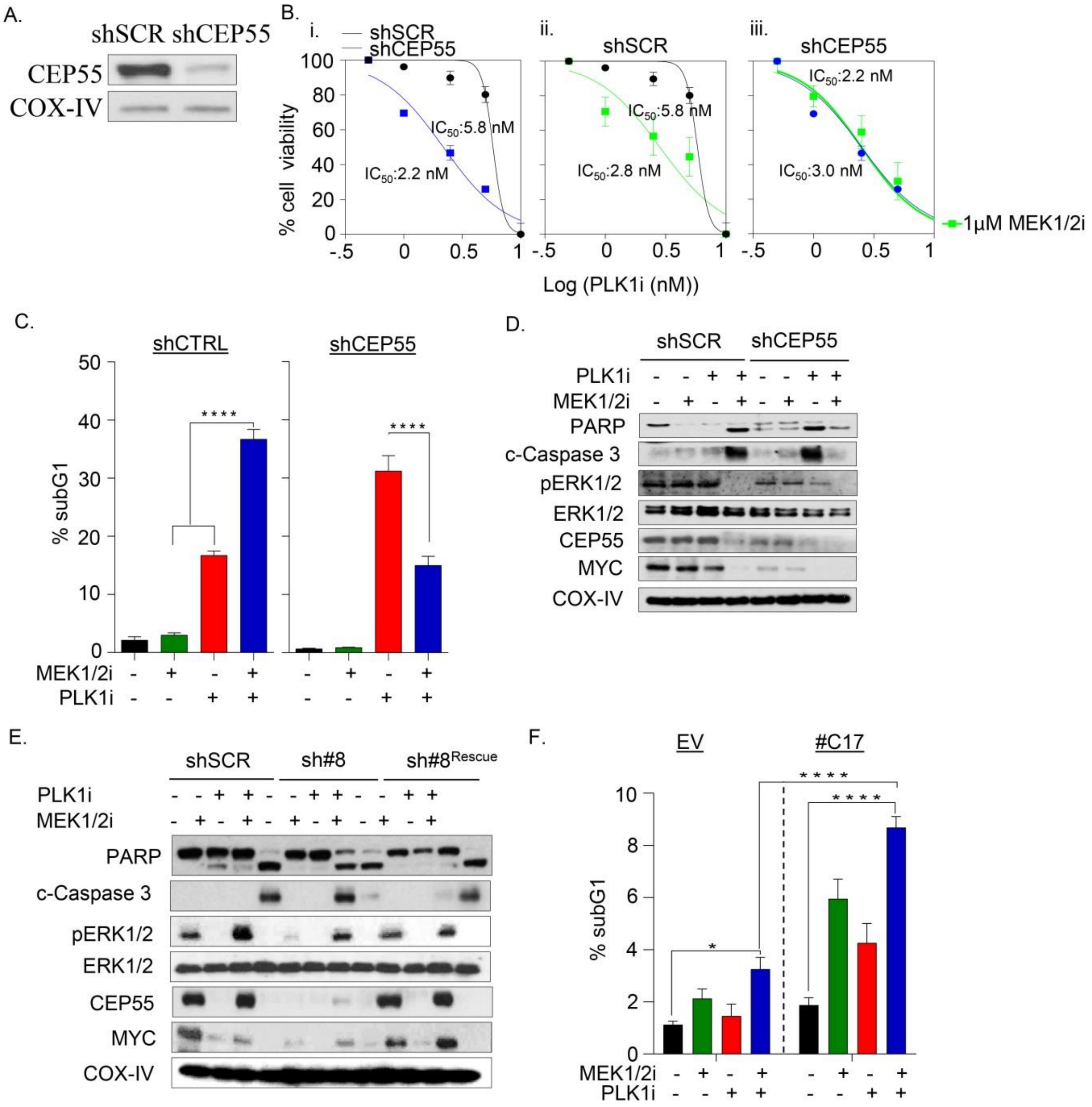

G.

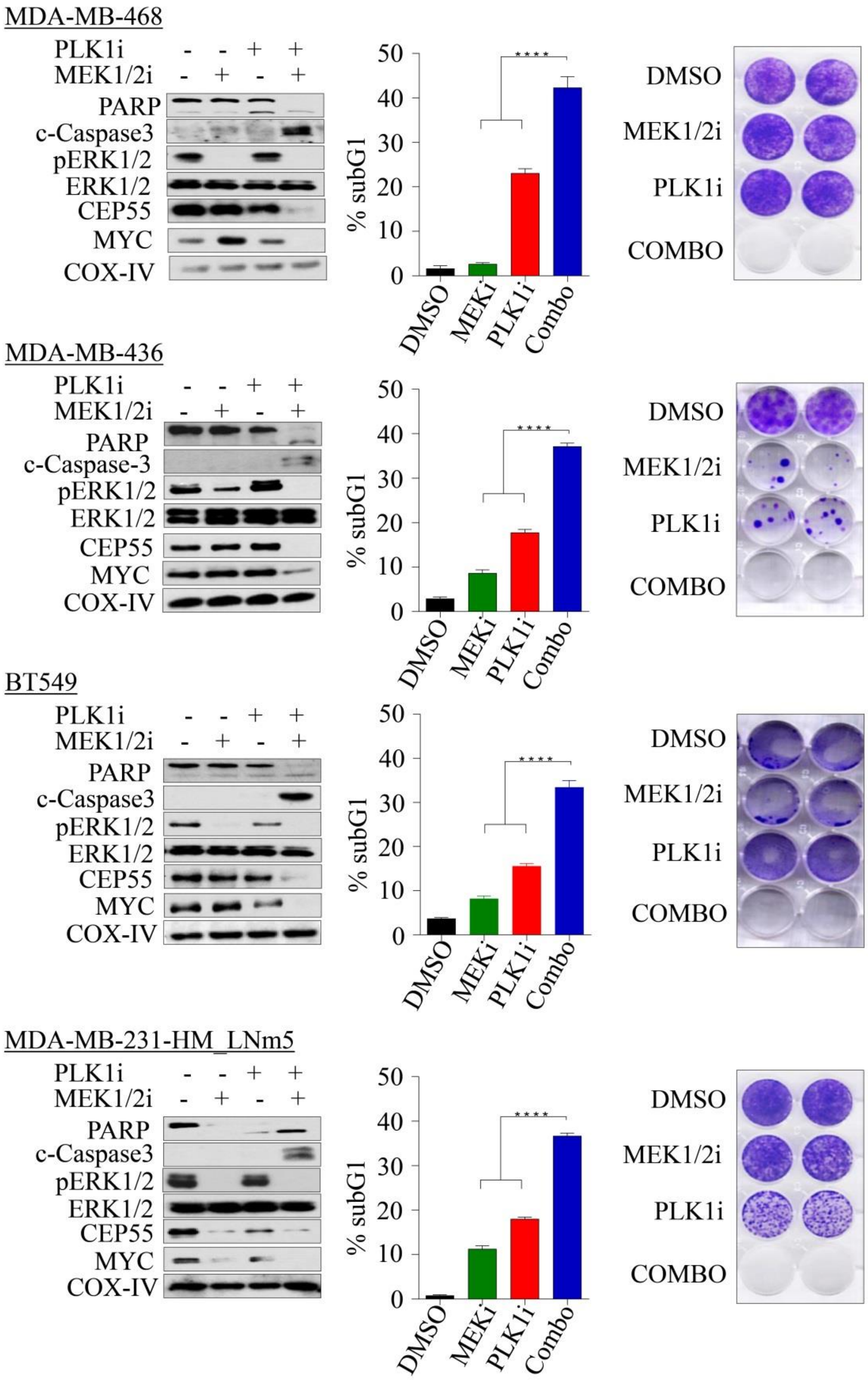

MDA-MB-468
PLK1i - - + +
MEK1/2i - + - +
PARP
c-Caspase3
pERK1/2
ERK1/2
CEP55
MYC
COX-IV
% subG1
0 10 20 30 40 50
****
DMSO
MEKi
PLK1i
Combo
DMSO
MEK1/2i
PLK1i
COMBO
MDA-MB-436
PLK1i - - + +
MEK1/2i - + - +
PARP
c-Caspase-3
pERK1/2
ERK1/2
CEP55
MYC
COX-IV
% subG1
0 10 20 30 40 50
****
DMSO
MEKi
PLK1i
Combo
DMSO
MEK1/2i
PLK1i
COMBO
BT549
PLK1i - - + +
MEK1/2i - + - +
PARP
c-Caspase3
pERK1/2
ERK1/2
CEP55
MYC
COX-IV
% subG1
0 10 20 30 40 50
****
DMSO
MEKi
PLK1i
Combo
DMSO
MEK1/2i
PLK1i
COMBO
MDA-MB-231-HM_LNm5
PLK1i - - + +
MEK1/2i - + - +
PARP
c-Caspase3
pERK1/2
ERK1/2
CEP55
MYC
COX-IV
% subG1
0 10 20 30 40 50
****
DMSO
MEKi
PLK1i
Combo
DMSO
MEK1/2i
PLK1i
COMBO

4T1.2

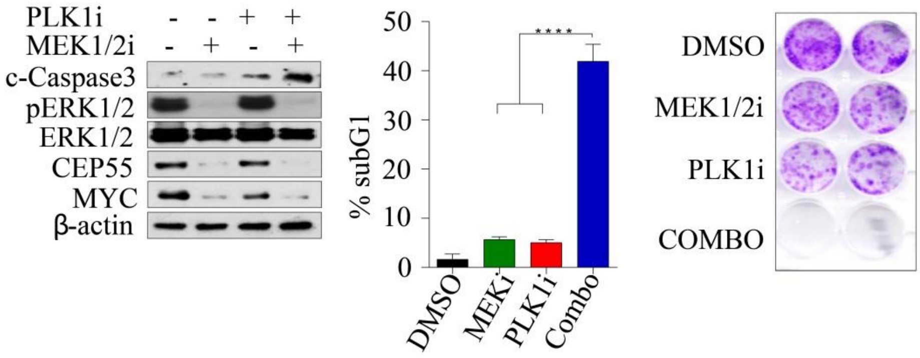

H.

MCF7

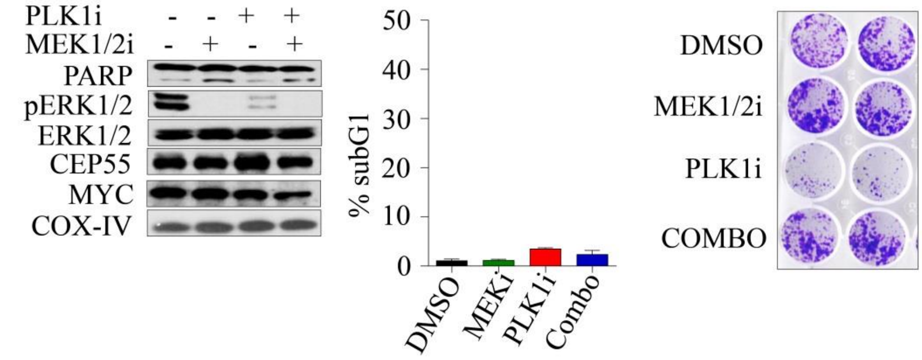

SKBR3

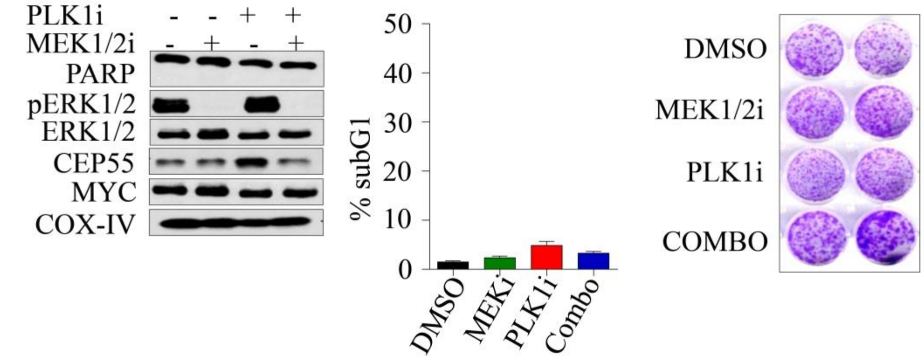

Appendix Figure S9

A.

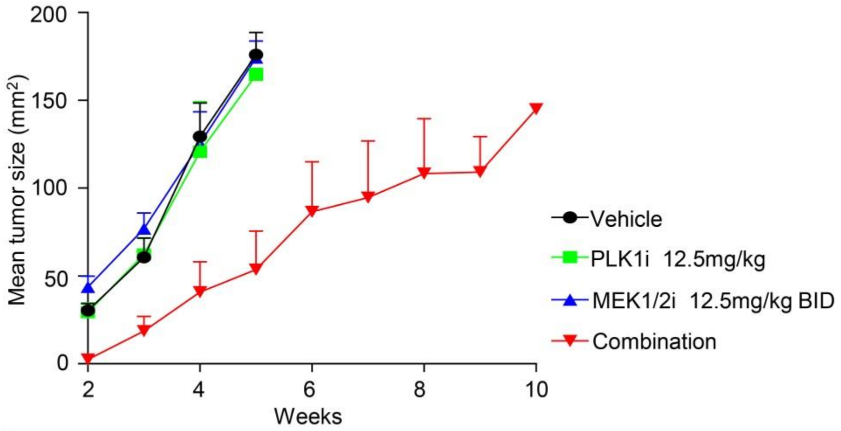

B.

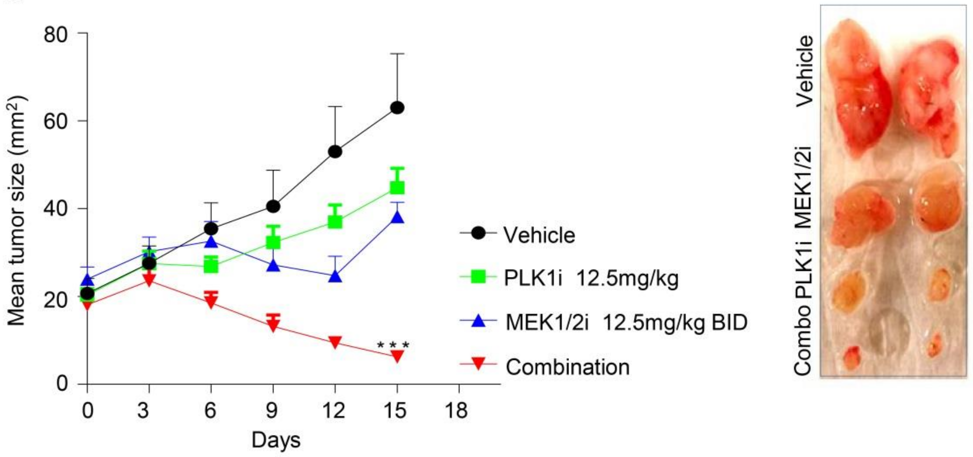

Supplement: Supplementary file 1 — Appendix [file EMMM-10-e8566-s001.pdf]
