# Supplementary material for: CEP55 is a determinant of cell fate during perturbed mitosis in breast cancer
Source: EMBO Mol Med. 2018 Aug 14;10(9):e8566. doi: 10.15252/emmm.201708566 (PMC6127888; doi:10.15252/emmm.201708566)
Supplement: Supplementary file 2 — Expanded View Figures PDF [file EMMM-10-e8566-s002.pdf]

Expanded View Figures

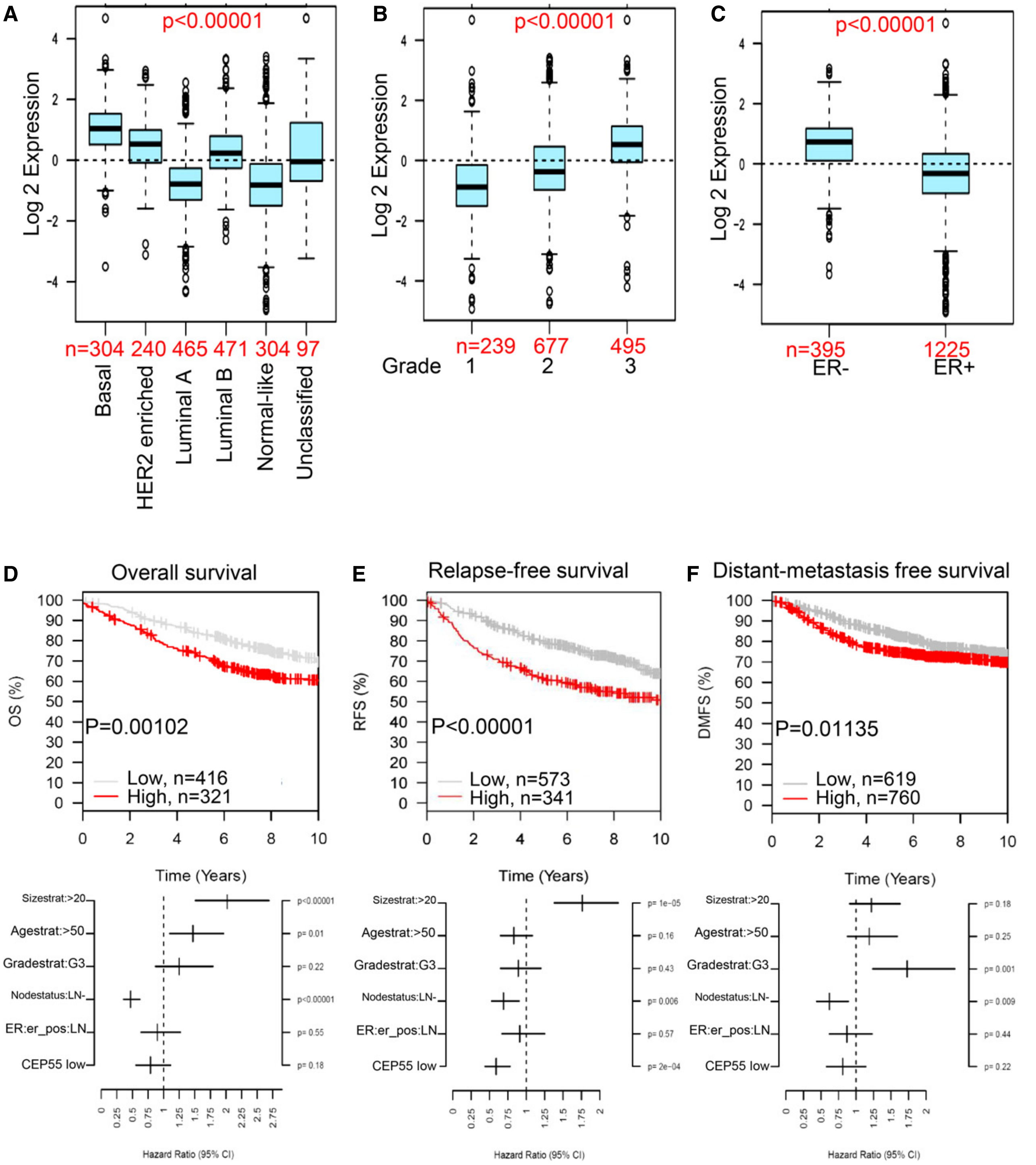

Figure EV1.

Figure EV1. Clinical correlation of CEP55 mRNA expression in breast cancer datasets.

A–C Relationship between *CEP55* mRNA expression (Log 2 expression) and (A) breast cancer intrinsic molecular subtypes, (B) histological grade, and (C) estrogen receptor (ER) status evaluated through the GOBO online tool (http://co.bmc.lu.se/gobo/; Ringner *et al*, 2011). Number of patients used for the analysis was indicated at the bottom of each panel.

D–F Top panel, association of *CEP55* expression with clinical outcome for overall survival (D), relapse-free survival (E) and distant metastasis-free survival (F) determined using the GOBO datasets; bottom panel, corresponding multivariate parameters analyses. Patients were divided into *CEP55* low and high expression.

Figure EV2. CEP55 regulates human breast cancer cell survival (related to Fig 1).

A Immunoblot analysis of CEP55 expression showing knockdown efficiency following 5 nM of pool siRNAs transfection at 72 h in representative triple-negative breast cancer lines. COX-IV as a loading control.

B Effect of CEP55 knockdown using siCEP55 (5 nM) on cell proliferation and death in both BT549 and MDA-MB-436, triple-negative cell lines. Proliferation was assessed using the IncuCyte ZOOM® live-cell imager. The percentage of cell confluence was determined using an IncuCyte mask analyzer (left panel), and apoptotic fractions (sub-G1 population) was determined by propidium iodide staining using flow cytometry and ModFit LT 4.0 software analysis (right panel). Graphs represent the mean ± SEM of two independent experiments. *$P \leq 0.05$; **$P \leq 0.01$; ***$P \leq 0.001$; ****$P \leq 0.0001$.

C Left, representative cytogram analysis of control and CEP55 knockdown MDA-MB-231 cells showing cell cycle profiles at day 30. Cells were exposed to doxycycline (2 μg/ml) twice weekly for 4 weeks. Middle, cell cycle distribution and right, sub-G1 analyses of corresponding data are shown. Graphs represent representative data from two independent experiments. *$P \leq 0.05$. Mean ± SEM.

D Sequencing chromatograms of both wild-type and shRNA-resistant constructs verified by Sanger sequencing are shown.

E Top, immunoblot analysis of CEP55 expression in CEP55 knockdown MDA-MB-231 cells. The shRNA-resistant construct was transiently transfected with 1 μg of DNA in sh#8 cells, and CEP55 expression was determined after 48 h post-transfection. COX-IV served as loading control. Bottom, effect of CEP55 reintroduction on cell proliferation in CEP55-knockdown sh#8 cells assessed using the IncuCyte ZOOM® live-cell imager as described in panel (B). Graph represents the mean ± SEM of three independent experiments.

F Quantification of crystal violet intensity staining (absorbance value at 540 nM) on colony-forming capacity in both control and CEP55 knockdown in MDA-MB-231 cells. For colony formation assay, 1,000 cells were seeded. ****$P \leq 0.0001$. Mean ± SEM.

© 2018 The Authors

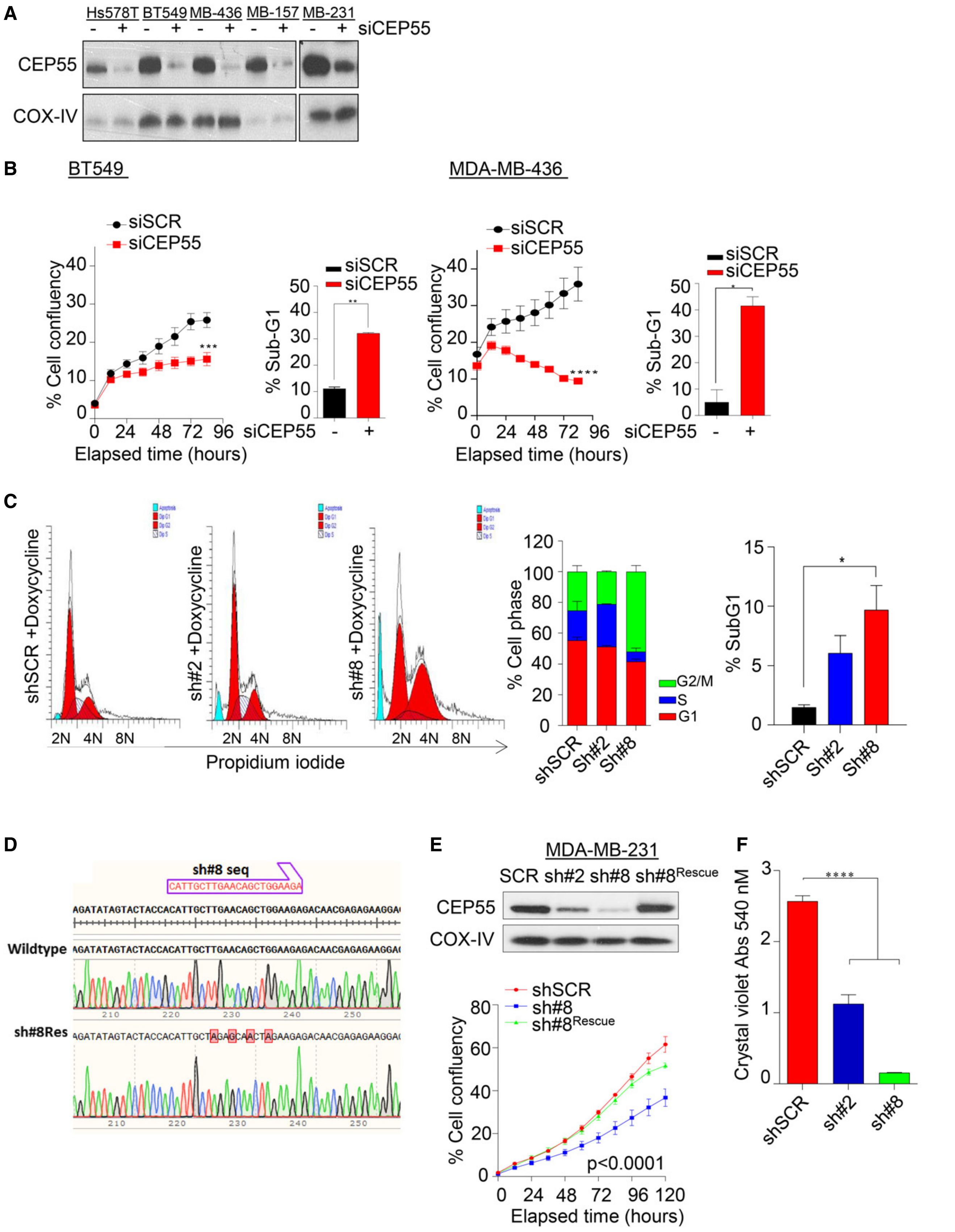
A
Hs578T BT549 MB-436 MB-157 MB-231
siCEP55
CEP55
COX-IV
B
BT549
MDA-MB-436
siSCR
siCEP55
% Cell confluency
Elapsed time (hours)
% Sub-G1
C
shSCR +Doxycycline
sh#2 +Doxycycline
sh#8 +Doxycycline
Propidium iodide
% Cell phase
G2/M
S
G1
% SubG1
shSCR
Sh#2
Sh#8
D
sh#8 seq
CATTGCTTGAACAGCTGGAAGA
Wildtype
sh#8Res
E
MDA-MB-231
SCR sh#2 sh#8 sh#8Rescue
shSCR
sh#8
sh#8Rescue
p<0.0001
F
Crystal violet Abs 540 nM
shSCR
sh#2
sh#8

Figure EV2.

Figure EV3. CEP55 regulates genomic instability and aneuploidy (related to Fig 3).

A Immunoblot analysis of CEP55 expression in CEP55 knockdown Hs578T using constitutive shRNA construct. COX-IV as a loading control.
B Effect of CEP55 knockdown on cell proliferation in Hs578T cells assessed using IncuCyte ZOOM® live-cell imager phase-only processing module for 120 h. Percentage of cell confluence was determined using an IncuCyte mask analyzer. Graph represents the mean ± SEM of two independent experiments.
C Evaluation of colony-forming capacity at 14 days determined using crystal violet staining in both control and CEP55 knockdown Hs578T cells. For colony formation assay, 1,000 cells were seeded.
D Percentage of aneuploidy in both control and CEP55 knockdown Hs578T cells determined using ModFit LT 4.0 software. Yellow peaks represent subpopulation of aneuploidy. Graph represents the mean ± SEM of two independent experiments. ****$P \leq 0.0001$.
E Representative SNP array plot showing chromosomes alteration following CEP55-knockdown in MDA-MB-231 cells (sh#2), matched with Fig 2C, $n = 2$.
F Comparison of percentage of genome altered evaluated using amplified copies (6–8 copies across genome) between parental MDA-MB-231 cells and its isogenic metastatic MDA-MB-231-HM-LNm5 cells determined using data from SNP arrays.
G Immunoblot analysis on CEP55 expression using *CEP55* shRNA construct in MDA-MB-231- HM-LNm5 metastatic line. Cells were transiently transfected with 1 μg of DNA, and knockdown of CEP55 levels was determined after 48 h post-transfection. COX-IV as a loading control.
H Representative corresponding SNP array plots showing chromosomes alteration in both control and CEP55 knockdown MDA-MB-231- HM-LNm5 cells.
I Percentage of genome altered evaluated using amplified copies (6–8 copies across genome) in both control and CEP55 knockdown MDA-MB-231- HM-LNm5 cells.
J Venn diagram showing number of genes that were particularly gained, more than three copies following CEP55 overexpression in MCF10A cells.
K Ingenuity pathway analysis (IPA) showing cellular functions of amplified genes (> three copies) in CEP55-overexpressing MCF10A cells.
L Transient overexpression of Flag-CEP55 (1 μg of DNA) in MCF10A for 7 days and generation of polyploidy was calculated using ModFit LT 4.0 software. Graph represents the mean ± SEM of two independent experiments. *$P \leq 0.05$.

© 2018 The Authors

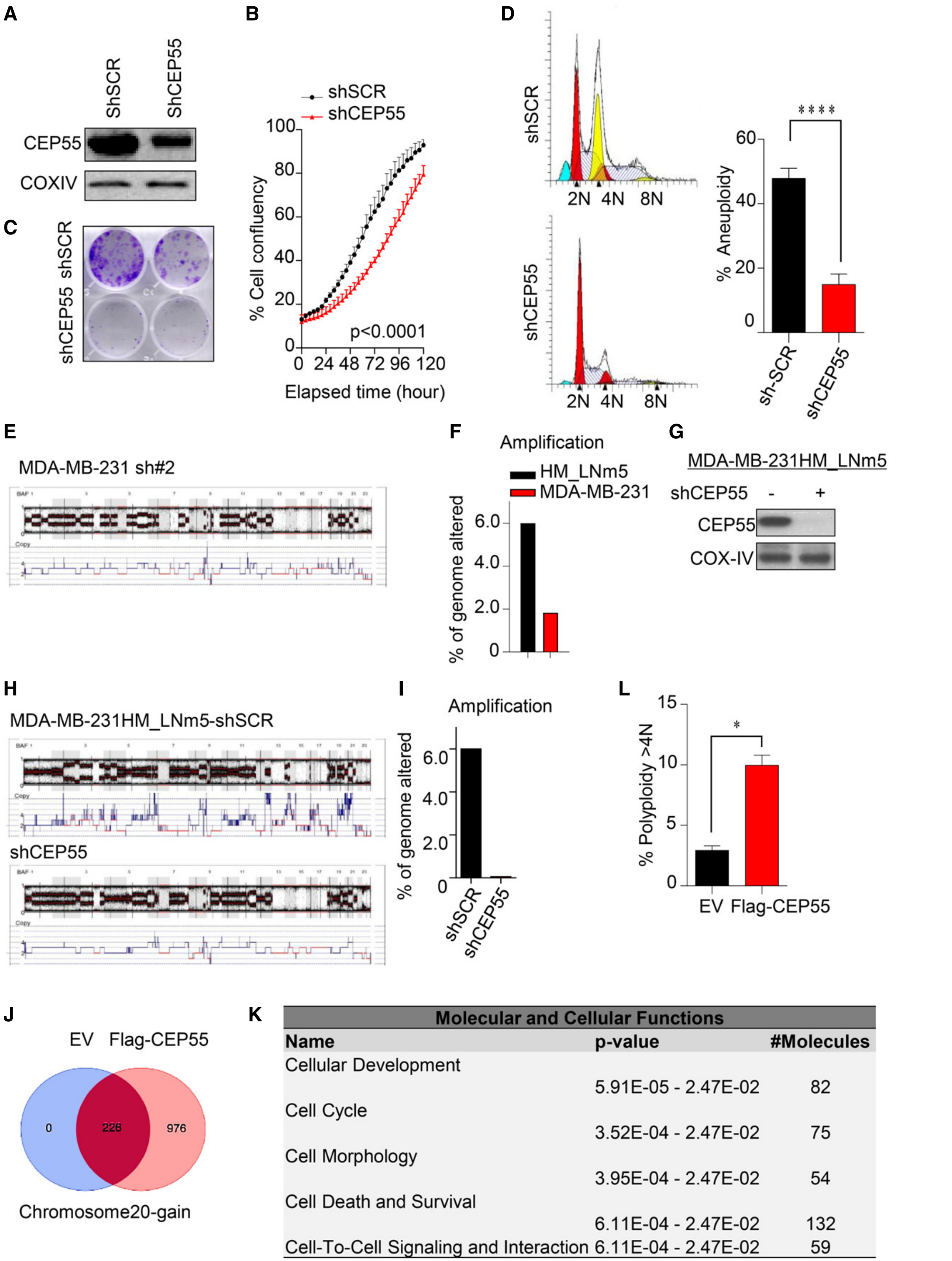

Molecular and Cellular Functions		
**Name**	**p-value**	**#Molecules**
Cellular Development	5.91E-05 - 2.47E-02	82
Cell Cycle	3.52E-04 - 2.47E-02	75
Cell Morphology	3.95E-04 - 2.47E-02	54
Cell Death and Survival	6.11E-04 - 2.47E-02	132
Cell-To-Cell Signaling and Interaction	6.11E-04 - 2.47E-02	59

Figure EV3.

Figure EV4. Overexpression of CEP55 rescues cell death during perturbed mitosis (related to Fig 4).

A, B Average time spent in mitosis (A) and mitotic outcomes (B) in empty vector (EV) and CEP55-overexpressing MCF10A cells following treatment with the BI25365 (10 nM) inhibitor similar to Fig 3A and B. Graph represents the mean ± SEM of two independent experiments. ****$P \leq 0.0001$.

C, D Cells were synchronized by double-thymidine block and released into either BI2536 (5 nM) or nocodazole (0.5 μM) treatments; then, cell lysates were collected after 24 h. Expression of pro- and anti-apoptotic effectors was then determined using immunoblotting analysis with indicated antibodies. COX-IV served as a loading control.

E CEP55 knockdown cells were transiently transfected with 20 nM of indicated siRNAs for 48 h followed by nocodazole (0.5 μM) treatment and immunoblotting as described in panel (C).

F, G Cells were synchronized by double-thymidine block and released into either BI2536 (5 nM) or nocodazole (0.5 μM) treatments; then, cell lysates were collected after 24 h. Expression of pro- and anti-apoptotic effectors was then determined using immunoblotting analysis with indicated antibodies. COX-IV served as a loading control.

 © 2018 The Authors

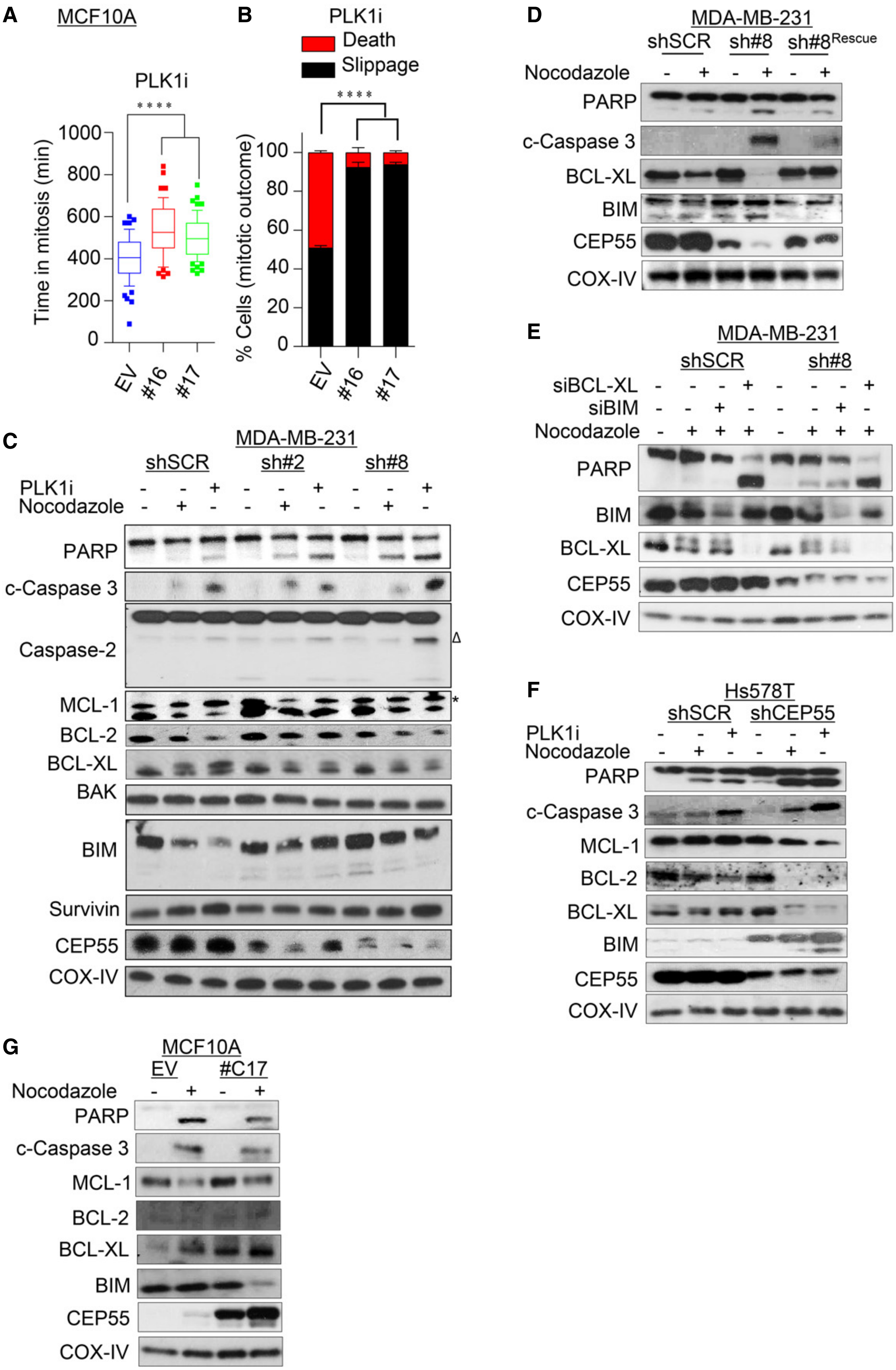
A
MCF10A
PLK1i
****
Time in mitosis (min)
0
200
400
600
800
1000
EV
#16
#17
B
PLK1i
Death
Slippage
****
% Cells (mitotic outcome)
0
20
40
60
80
100
EV
#16
#17
C
MDA-MB-231
shSCR
sh#2
sh#8
PLK1i
Nocodazole
PARP
c-Caspase 3
Caspase-2
MCL-1
BCL-2
BCL-XL
BAK
BIM
Survivin
CEP55
COX-IV
D
MDA-MB-231
shSCR
sh#8
sh#8Rescue
Nocodazole
PARP
c-Caspase 3
BCL-XL
BIM
CEP55
COX-IV
E
MDA-MB-231
shSCR
sh#8
siBCL-XL
siBIM
Nocodazole
PARP
BIM
BCL-XL
CEP55
COX-IV
F
Hs578T
shSCR
shCEP55
PLK1i
Nocodazole
PARP
c-Caspase 3
MCL-1
BCL-2
BCL-XL
BIM
CEP55
COX-IV
G
MCF10A
EV
#C17
Nocodazole
PARP
c-Caspase 3
MCL-1
BCL-2
BCL-XL
BIM
CEP55
COX-IV

Figure EV4.

Figure EV5. CEP55 overexpression mediated resistance to docetaxel treatment.

A Immunoblot analysis showing of cleaved caspase-3 and PARP in control and CEP55 knockdown MDA-MB-231 cells treated with either docetaxel (2.5 nM) or doxorubicin (30 nM) for 48 h. COX-IV served as a loading control.

B, C Effect of 1.0 nM (B) and 0.5 nM (C) of docetaxel treatment in both control and CEP55 knockdown MDA-MB-231 cells on cell proliferation assessed using IncuCyte ZOOM® live-cell imager phase-only processing module for 120 h. Percentage of cell confluence was determined using an IncuCyte mask analyzer. Graph represents the mean ± SEM of two independent experiments.

D Effect of 0.5 nM docetaxel treatment on long-term colony-forming capacity determined using crystal violet staining after 14 days of post-treatment (left) and quantification of crystal violet intensity using 0.1M sodium citrate extraction buffer relative to individual control cells (right). The baseline proliferation defect was adjusted as presented. Graph represents the mean ± SEM of two independent experiments. $*P \leq 0.05$; $****P \leq 0.0001$.

E, F Kaplan–Meier survival analysis of the relationship between *CEP55* mRNA expression and clinical outcomes in breast cancer patients treated with chemotherapy using the KMplotter dataset (Gyorffy *et al*, 2010). *CEP55* expression stratified both overall survival (HR: 2.12) (GSE3494, $n = 29$; GSE1456, $n = 40$) and relapse-free survival (HR: 1.67) (GSE1456, $n = 40$; GSE16391, $n = 14$; GSE17907, $n = 37$; GSE19615, $n = 81$; GSE21653, $n = 123$; GSE31519, $n = 40$; GSE3494, $n = 33$ and GSE5327, $n = 57$) in these cases.

 © 2018 The Authors

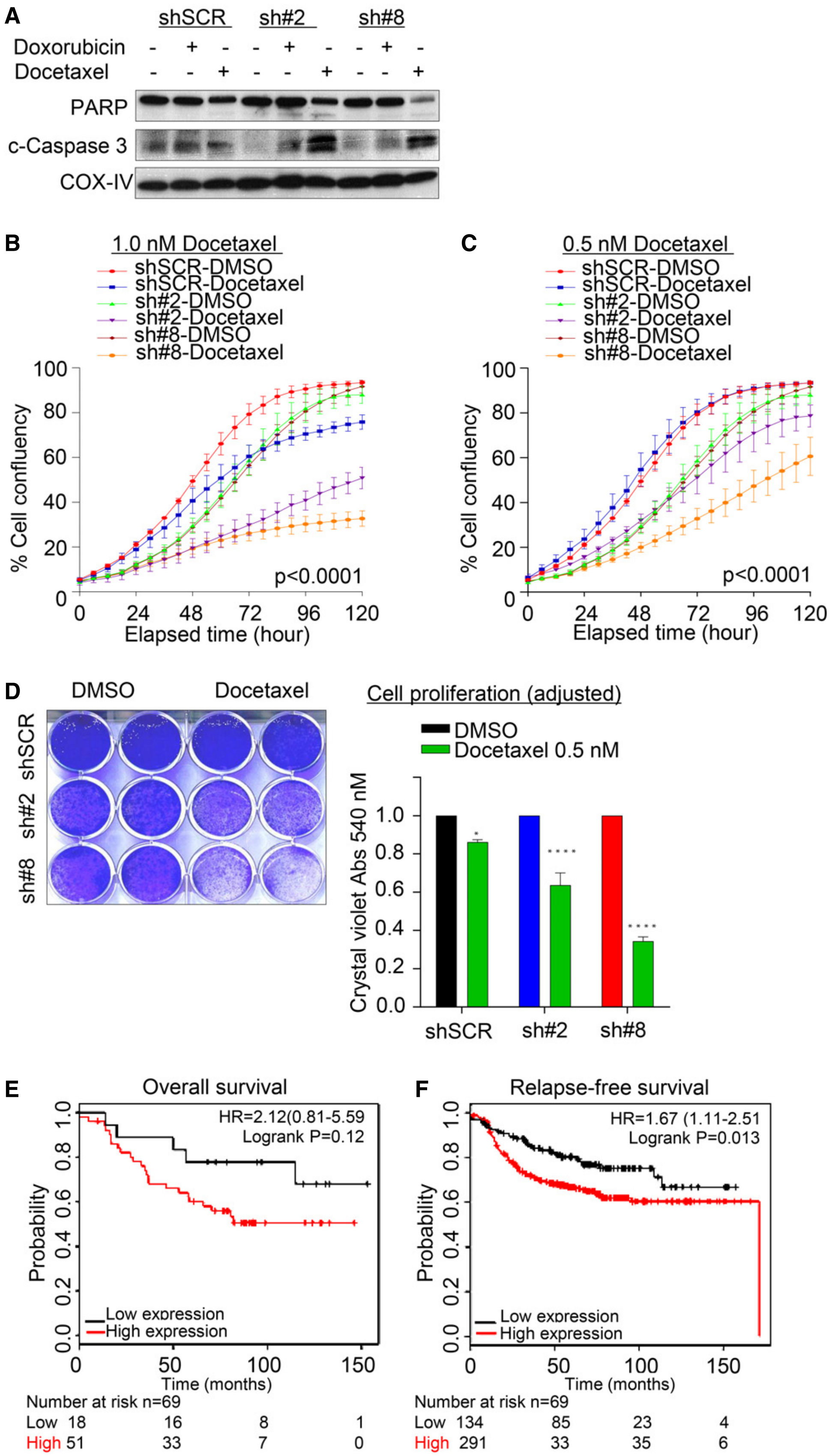
A
shSCR sh#2 sh#8
Doxorubicin - + - - + - - + -
Docetaxel - - + - - + - - +
PARP
c-Caspase 3
COX-IV
B
1.0 nM Docetaxel
shSCR-DMSO
shSCR-Docetaxel
sh#2-DMSO
sh#2-Docetaxel
sh#8-DMSO
sh#8-Docetaxel
% Cell confluency
p<0.0001
Elapsed time (hour)
C
0.5 nM Docetaxel
p<0.0001
D
DMSO Docetaxel
shSCR
sh#2
sh#8
Cell proliferation (adjusted)
DMSO
Docetaxel 0.5 nM
Crystal violet Abs 540 nM
E
Overall survival
HR=2.12(0.81-5.59
Logrank P=0.12
Probability
Low expression
High expression
Time (months)
Number at risk n=69
Low 18 16 8 1
High 51 33 7 0
F
Relapse-free survival
HR=1.67 (1.11-2.51
Logrank P=0.013
Number at risk n=69
Low 134 85 23 4
High 291 33 35 6

Figure EV5.
