# Supplementary material for: CEP55 is a determinant of cell fate during perturbed mitosis in breast cancer
Source: EMBO Mol Med. 2018 Aug 14;10(9):e8566. doi: 10.15252/emmm.201708566 (PMC6127888; doi:10.15252/emmm.201708566)
Supplement: Supplementary file 3 — Movie EV1 [file EMMM-10-e8566-s003.zip › EMM201708566MovieEV1/EMM-2017-08566-Movie-EV1/Movie_EV1.docx]

**Movie EV1:** Movie of MDA-MB-231 parental cell showing mitotic slippage upon PLK1 inhibition.
