# Supplementary material for: CEP55 is a determinant of cell fate during perturbed mitosis in breast cancer
Source: EMBO Mol Med. 2018 Aug 14;10(9):e8566. doi: 10.15252/emmm.201708566 (PMC6127888; doi:10.15252/emmm.201708566)
Supplement: Supplementary file 4 — Movie EV2 [file EMMM-10-e8566-s004.zip › EMM201708566MovieEV2/EMM-2017-08566-Movie-EV2/Movie_EV2.docx]

**Movies EV2 and EV3:** Movies of MDA-MB-231 shCEP55 knockdown cell showing mitotic cell death upon PLK1 inhibition.
