# Supplementary figures and images for: CEP55 is a determinant of cell fate during perturbed mitosis in breast cancer
Source: EMBO Mol Med. 2018 Aug 14;10(9):e8566. doi: 10.15252/emmm.201708566 (PMC6127888; doi:10.15252/emmm.201708566)

Figure EV8A

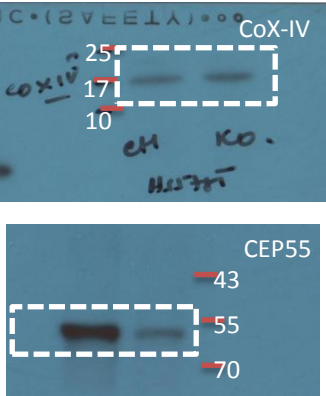

Figure EV8D

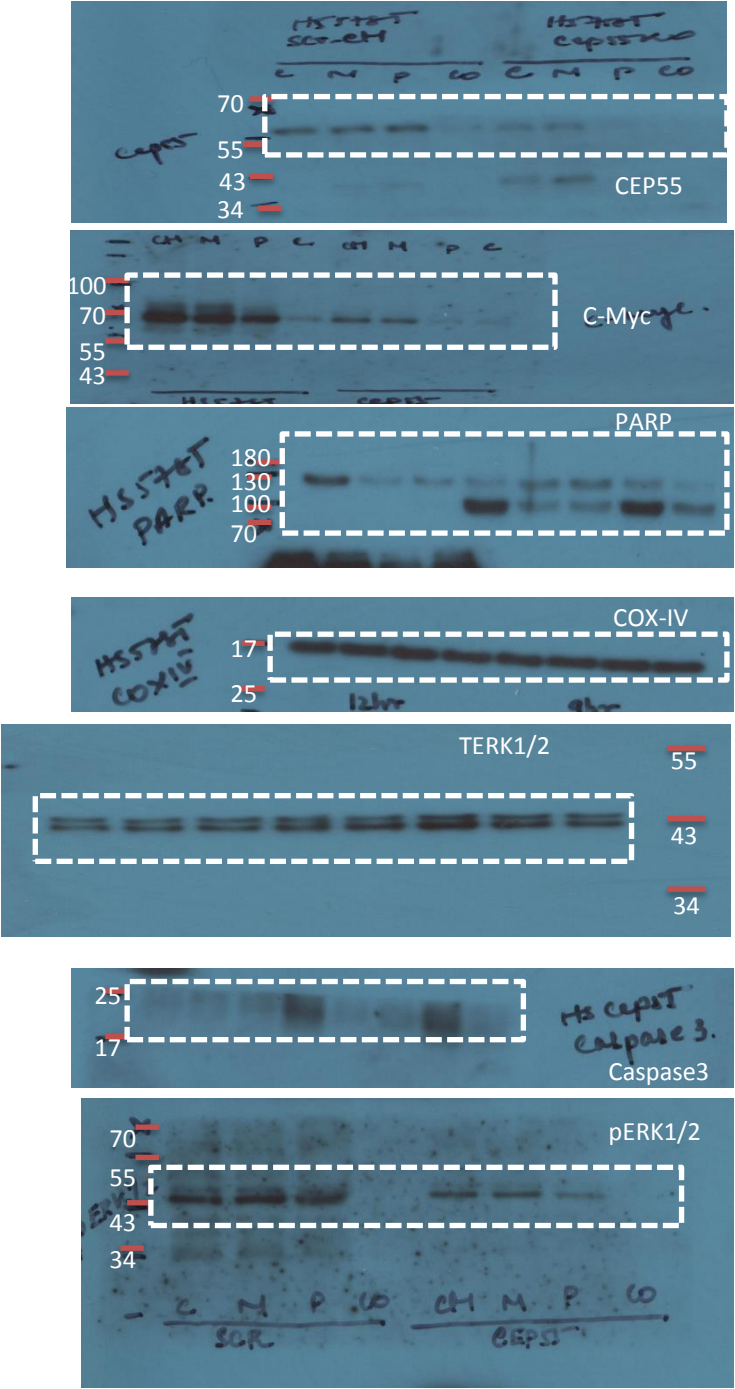

Figure EV8E

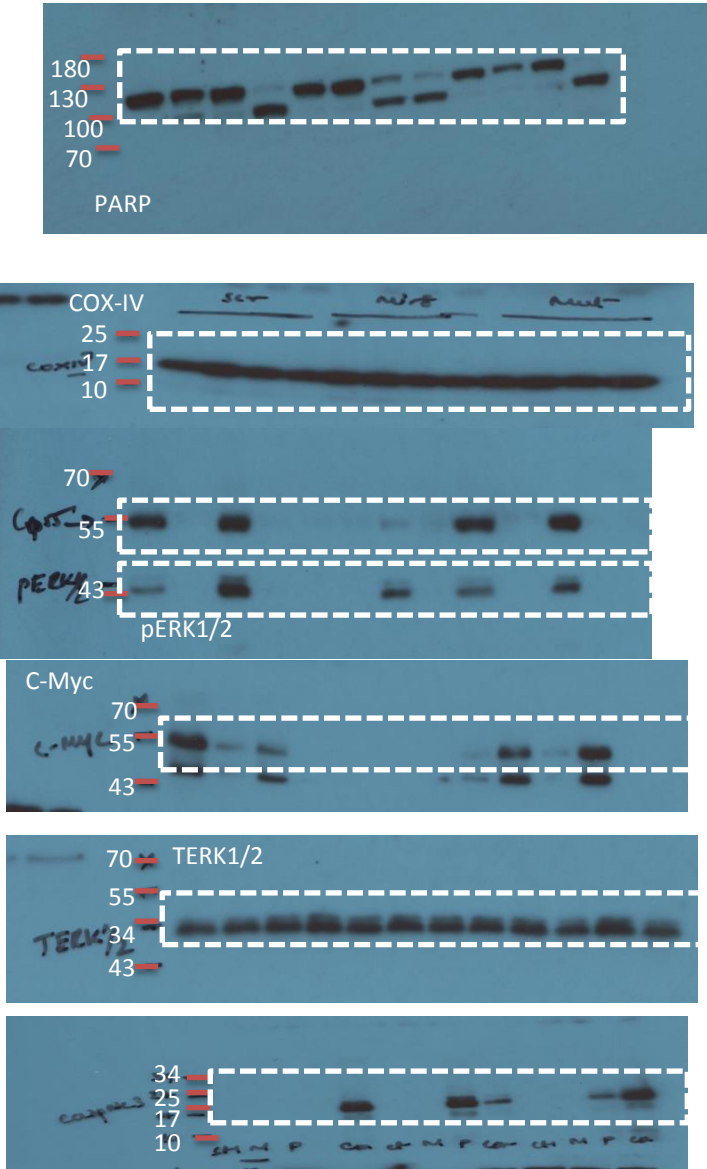

# MDA-MB-468

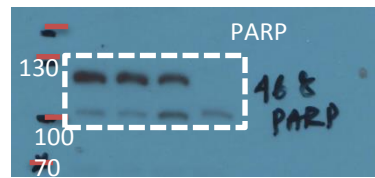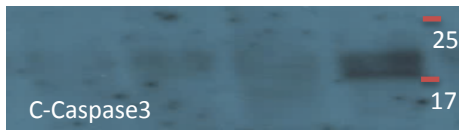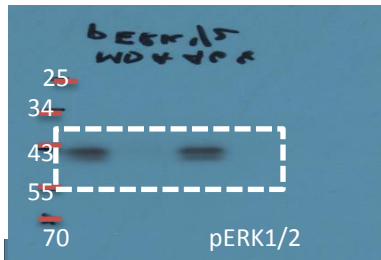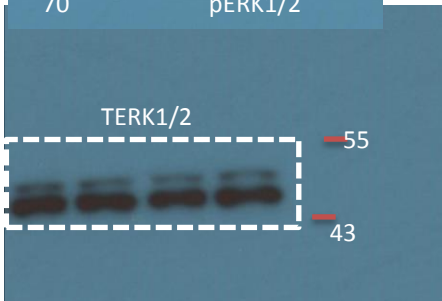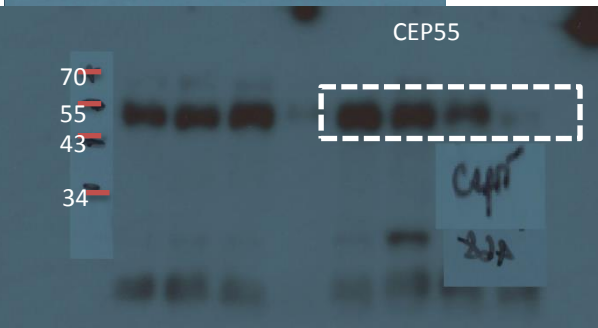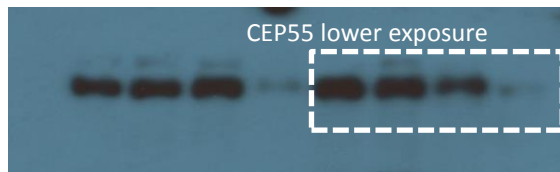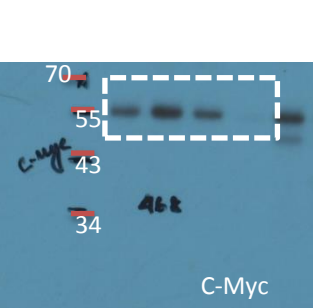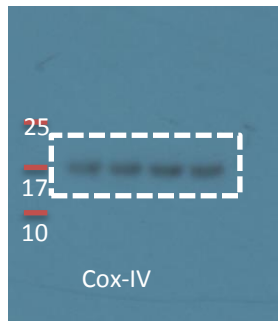

# MDA-MB-436

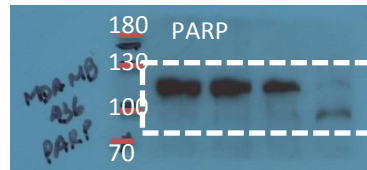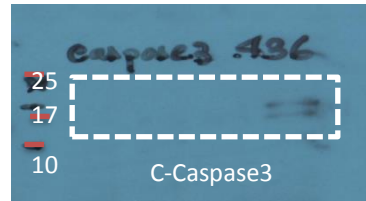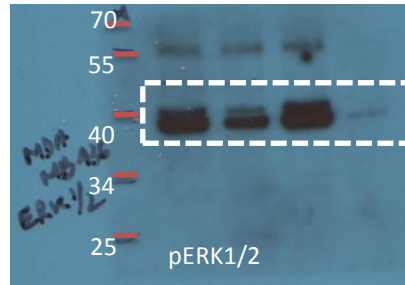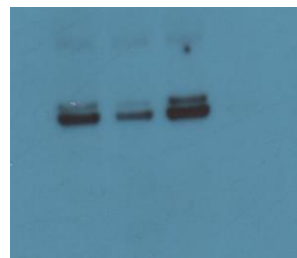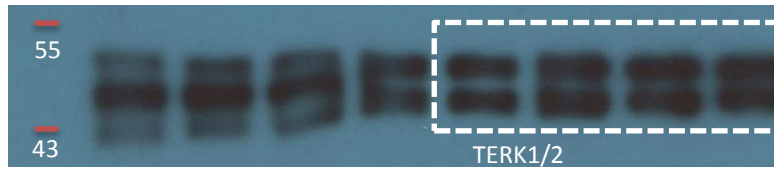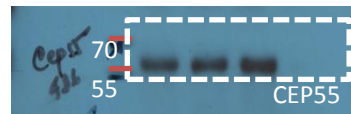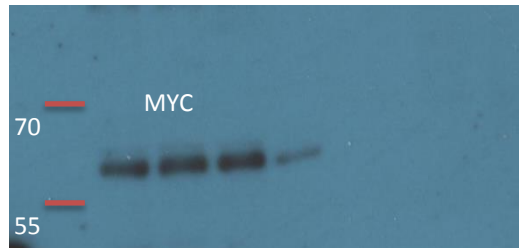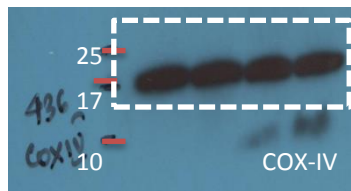

Figure EV8G

4T1.2

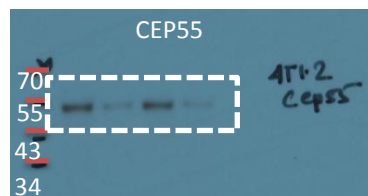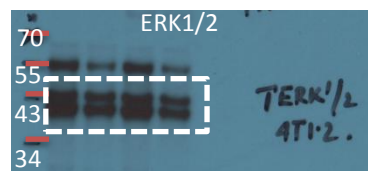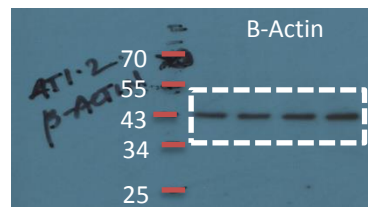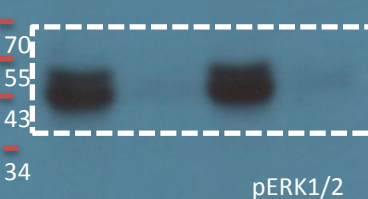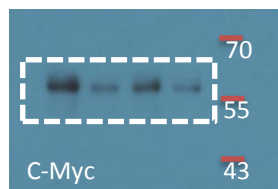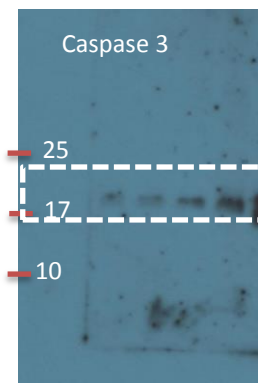

BT549

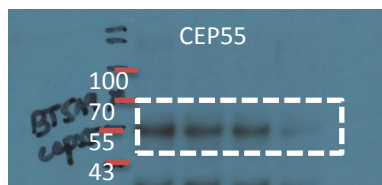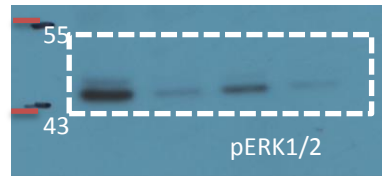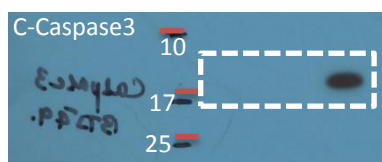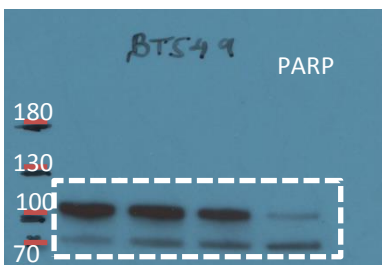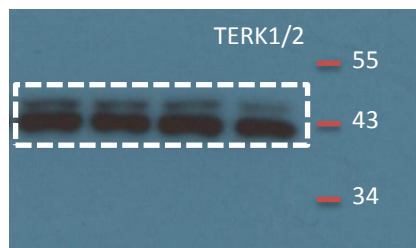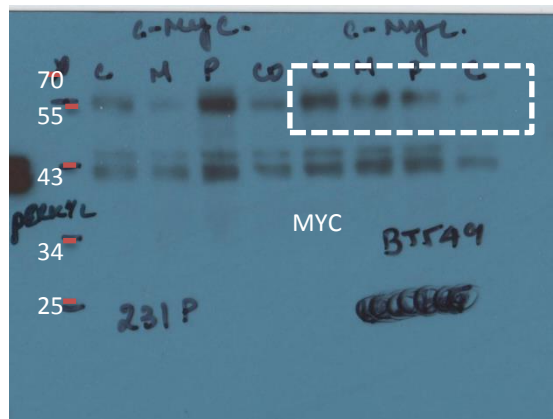

HM\_LNM5

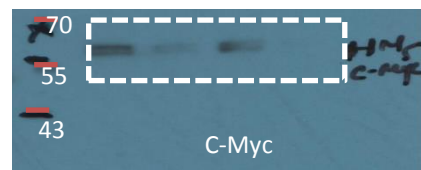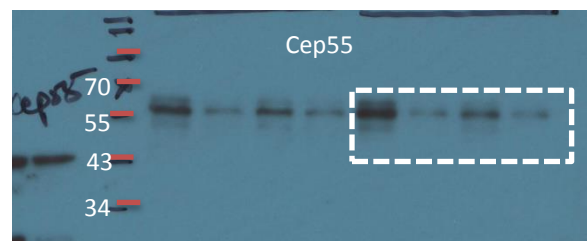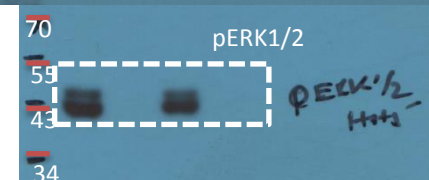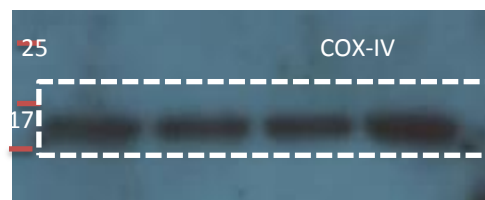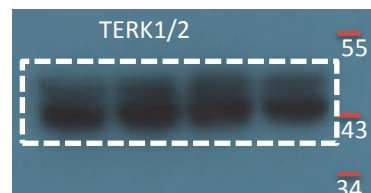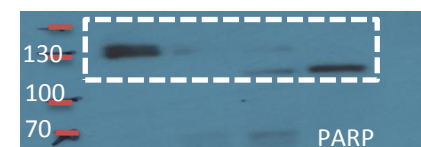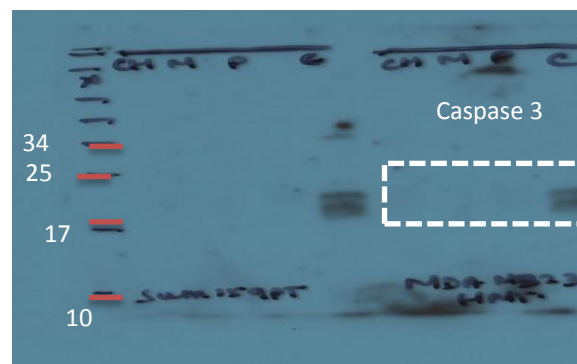

# SKBR3

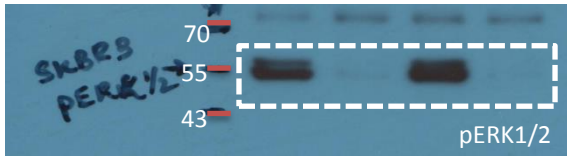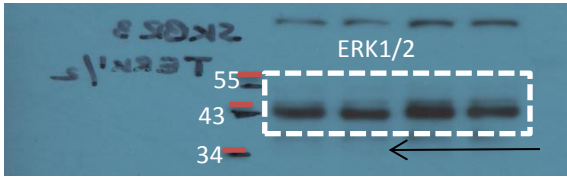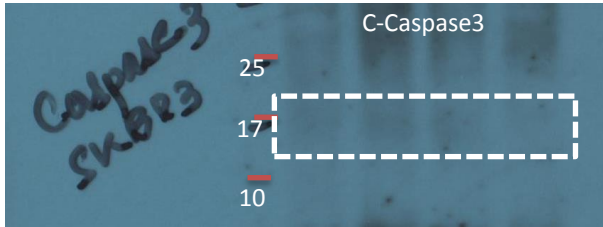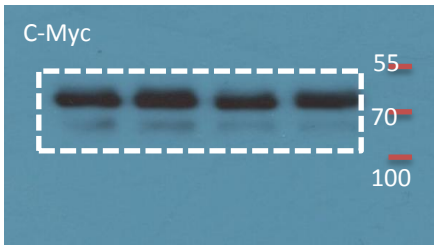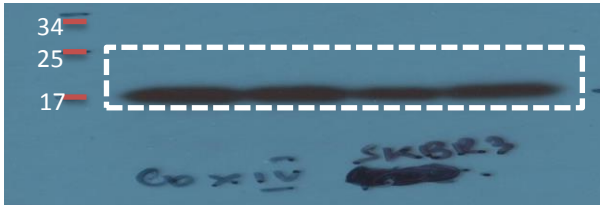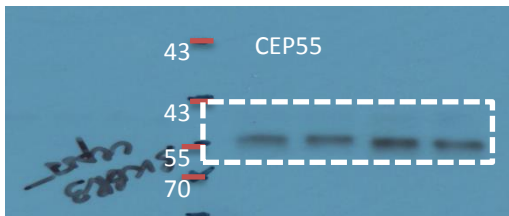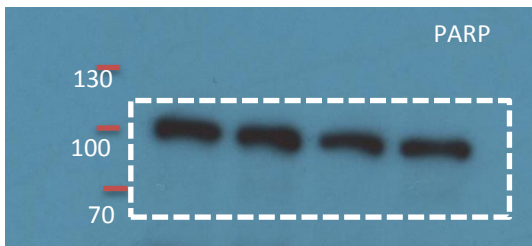

# MCF7

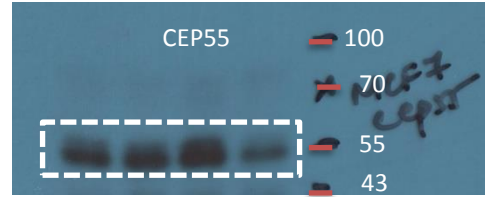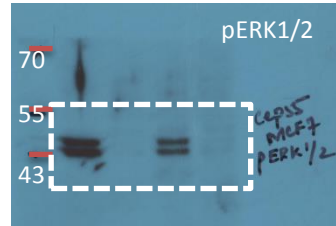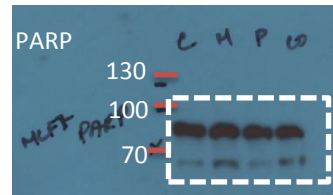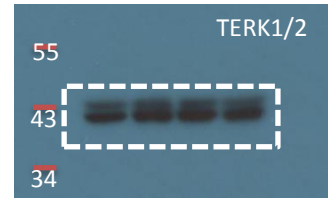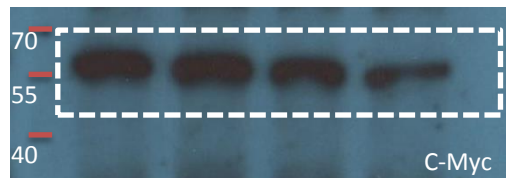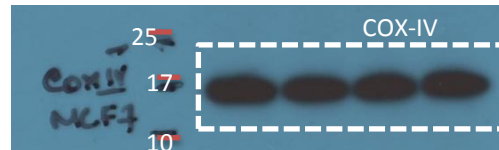

Supplement: Supplementary file 6 — Source Data for Expanded View [file EMMM-10-e8566-s009.zip › EMM201708566_source_data_AppendixFigS8.pdf]

Figure 4E

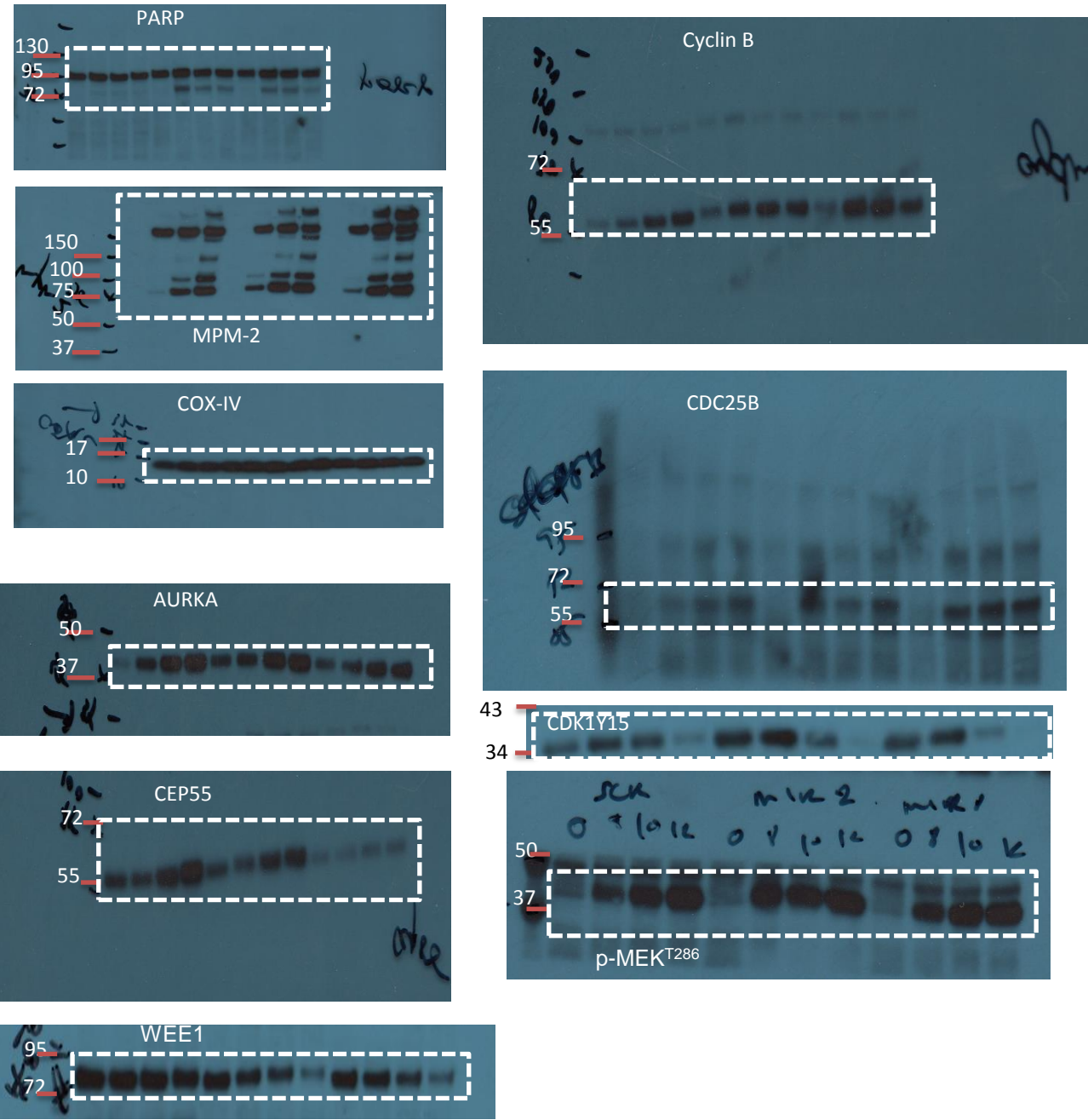

Supplement: Supplementary file 8 — Source Data for Figure 4 [file EMMM-10-e8566-s006.pdf]

Figure 5A

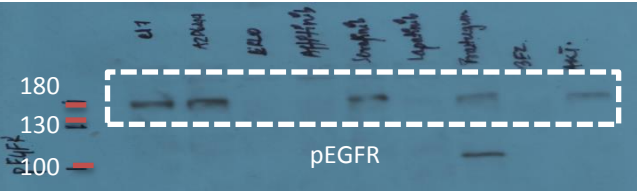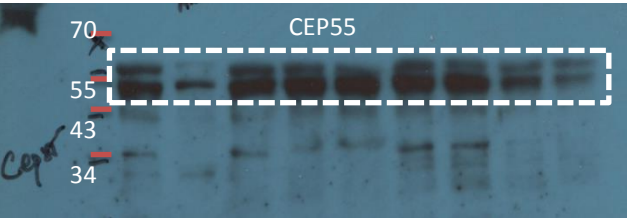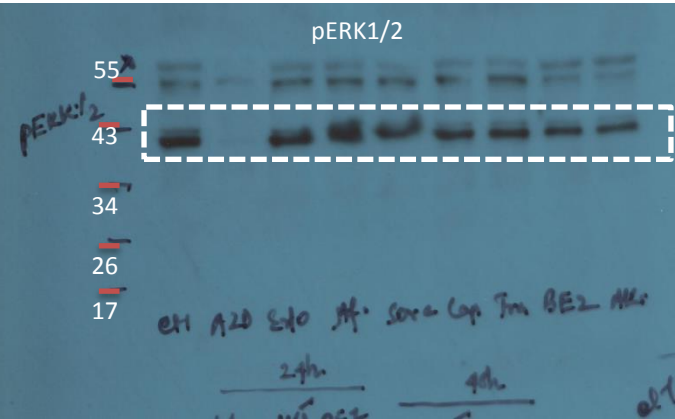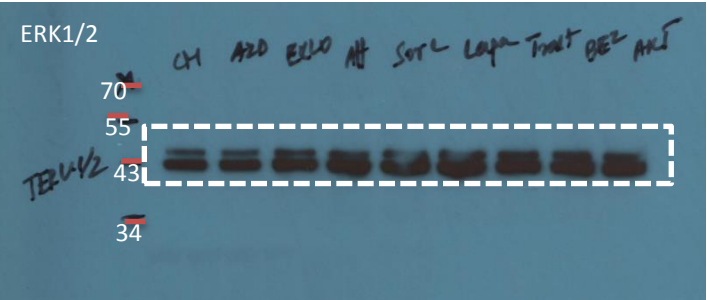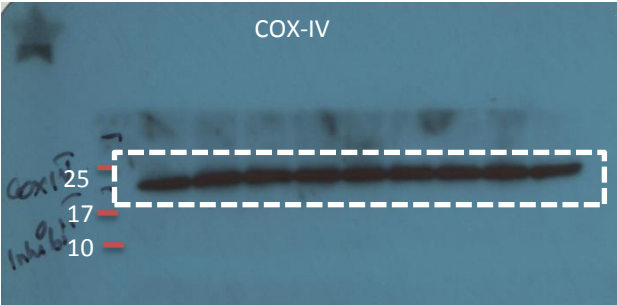

Figure 5B

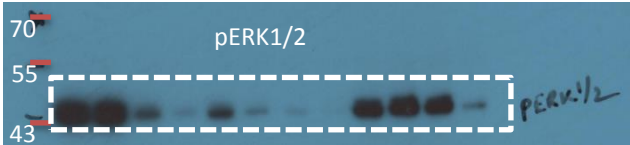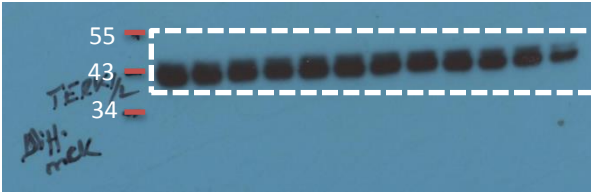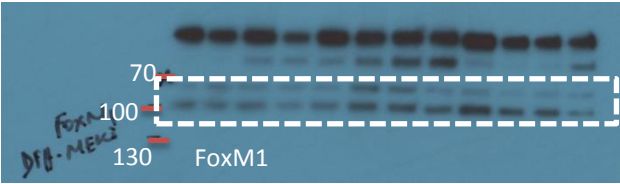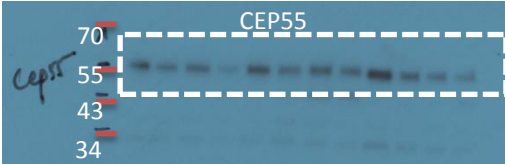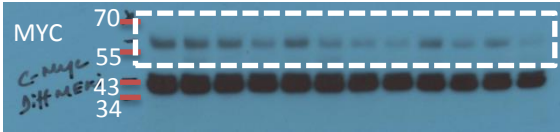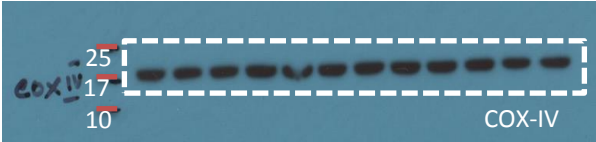

Figure 5D

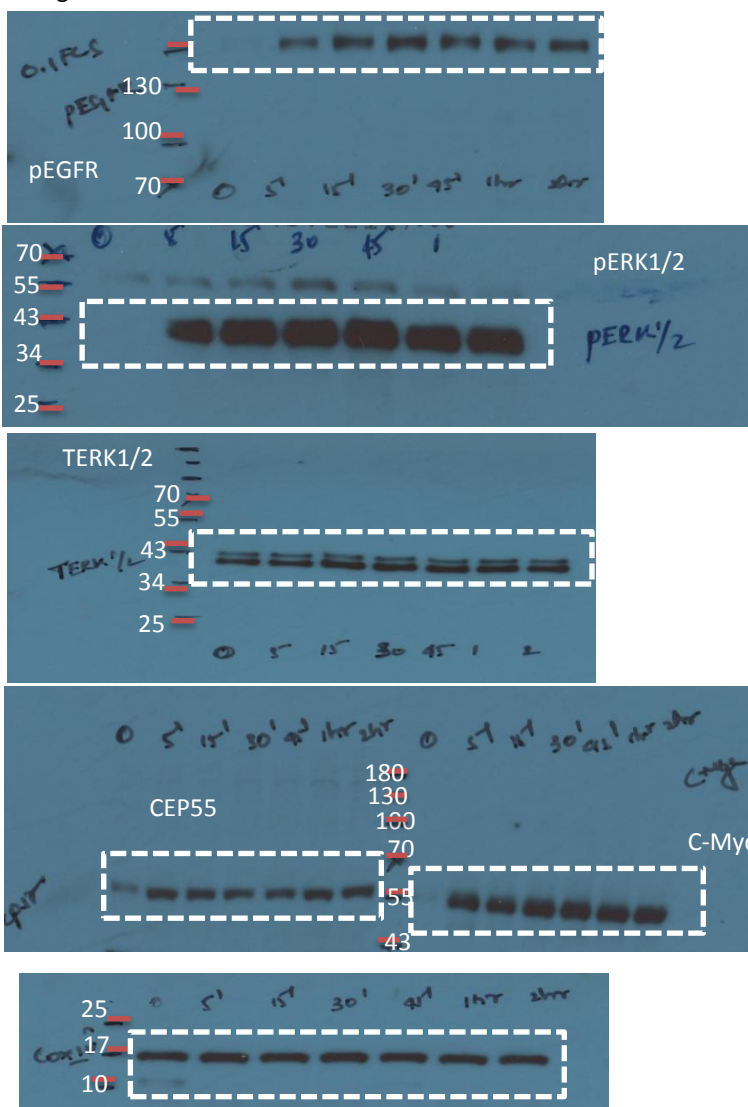

Figure 5F

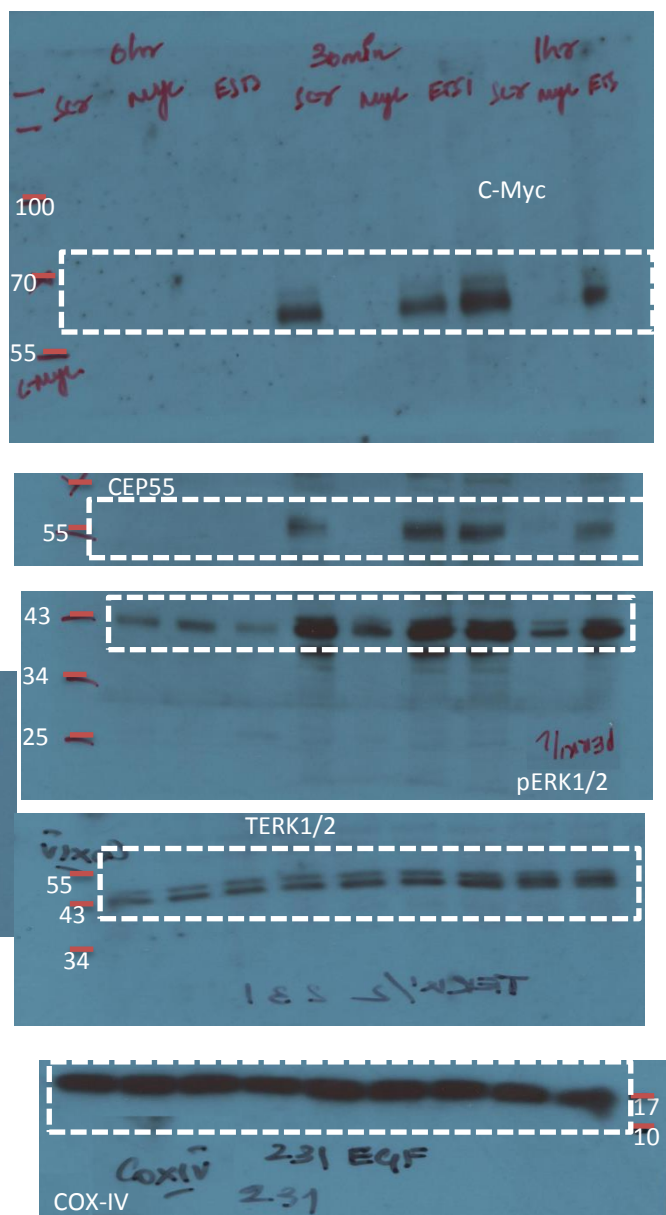

Figure 5G

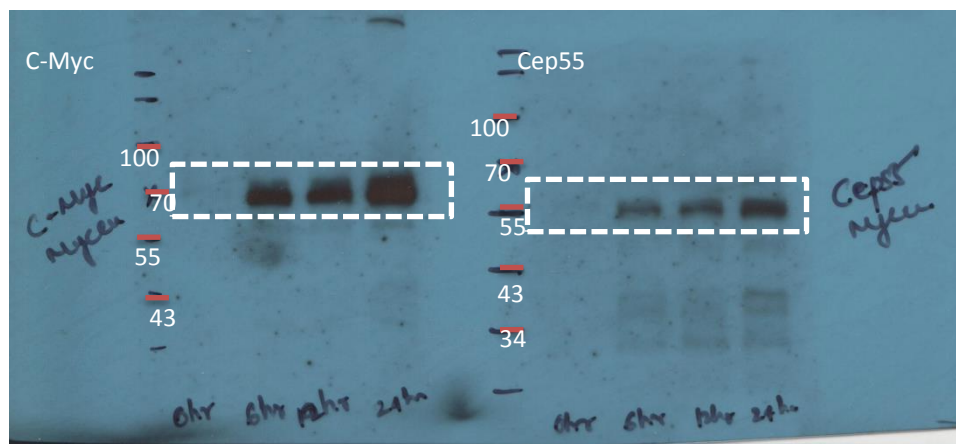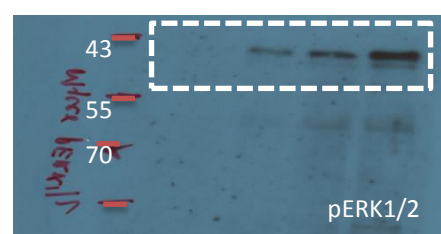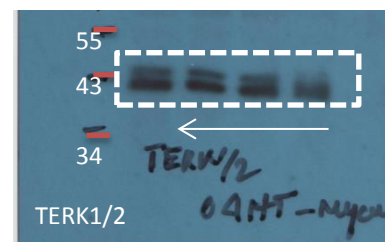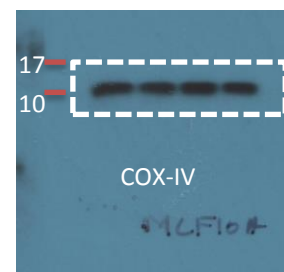

Figure 5L

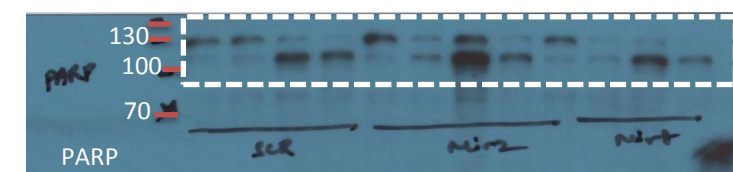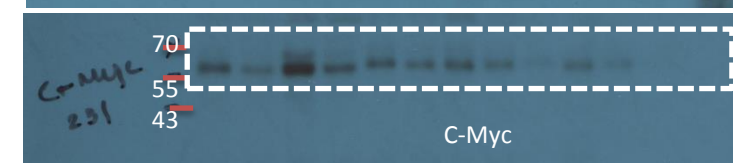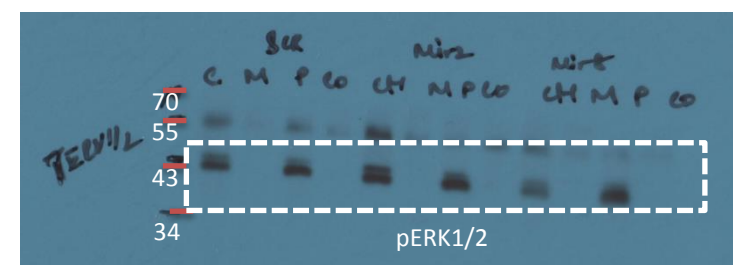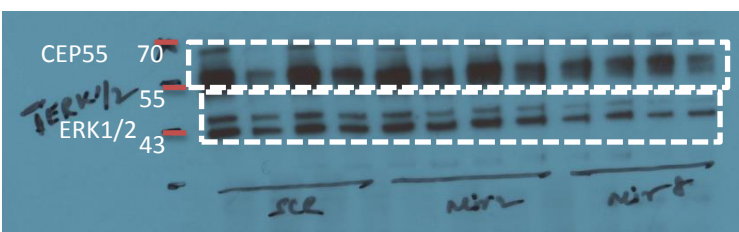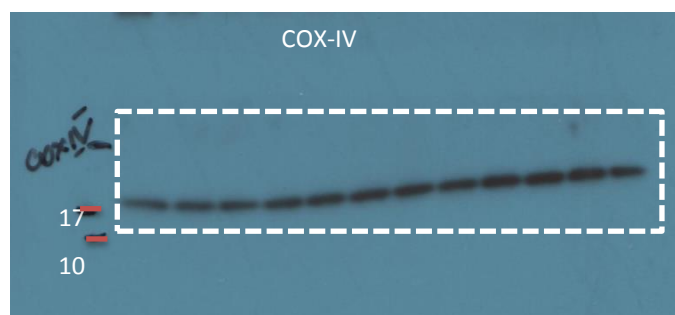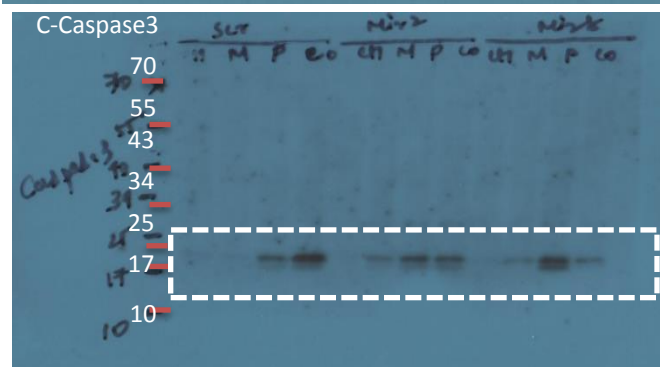

Supplement: Supplementary file 9 — Source Data for Figure 5 [file EMMM-10-e8566-s007.pdf]

Figure 6B

MDA-MB-157

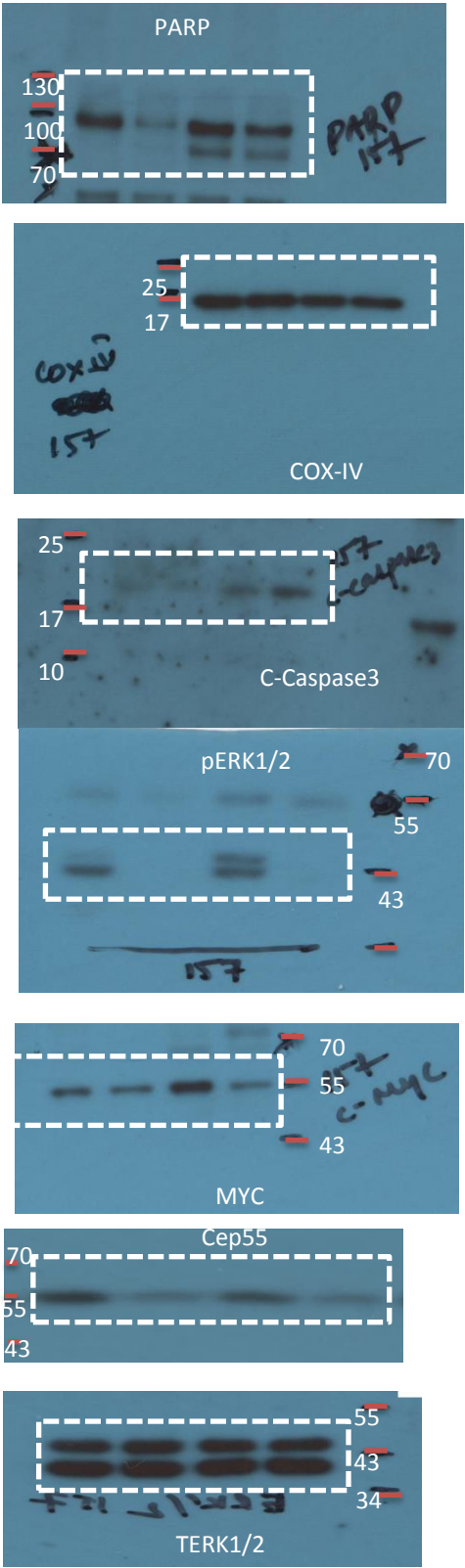

SUM159PT

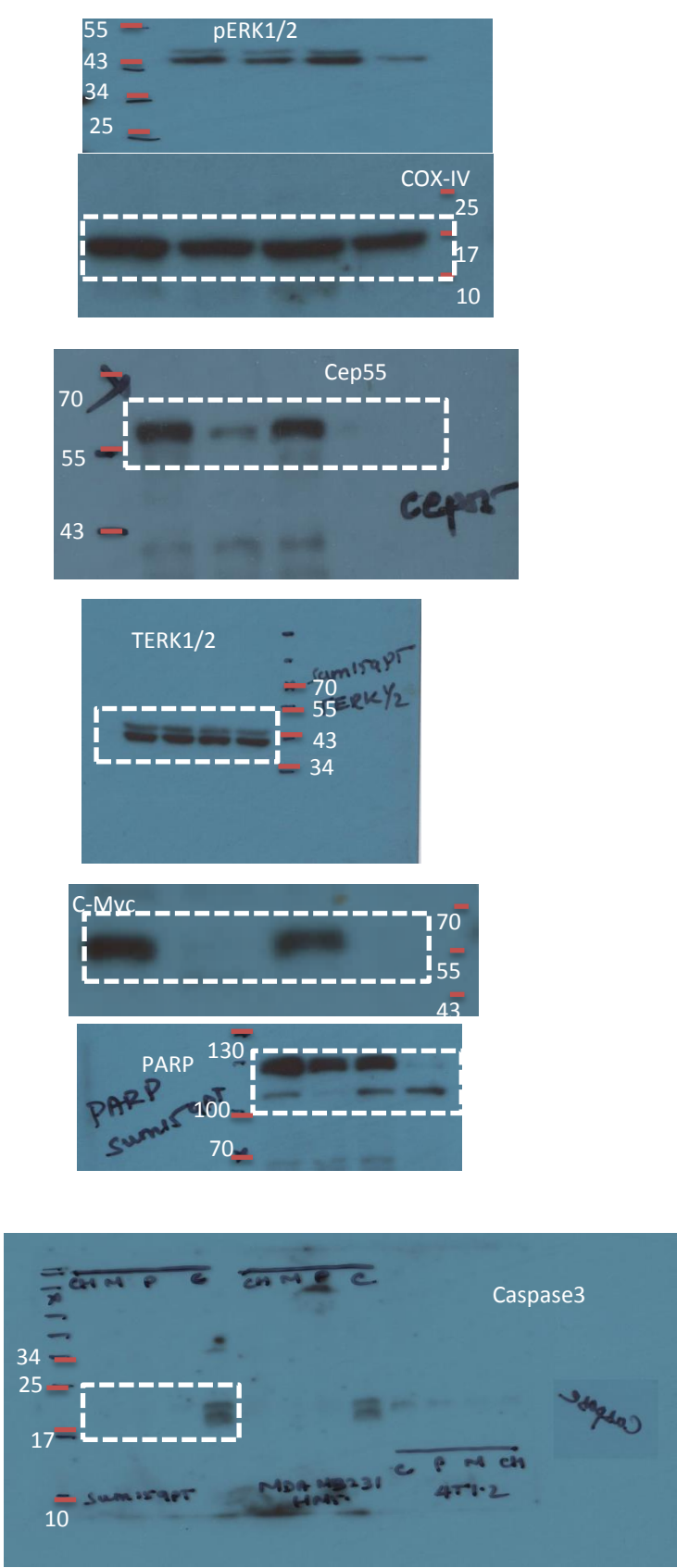

Figure 6D

MDA-MB-231

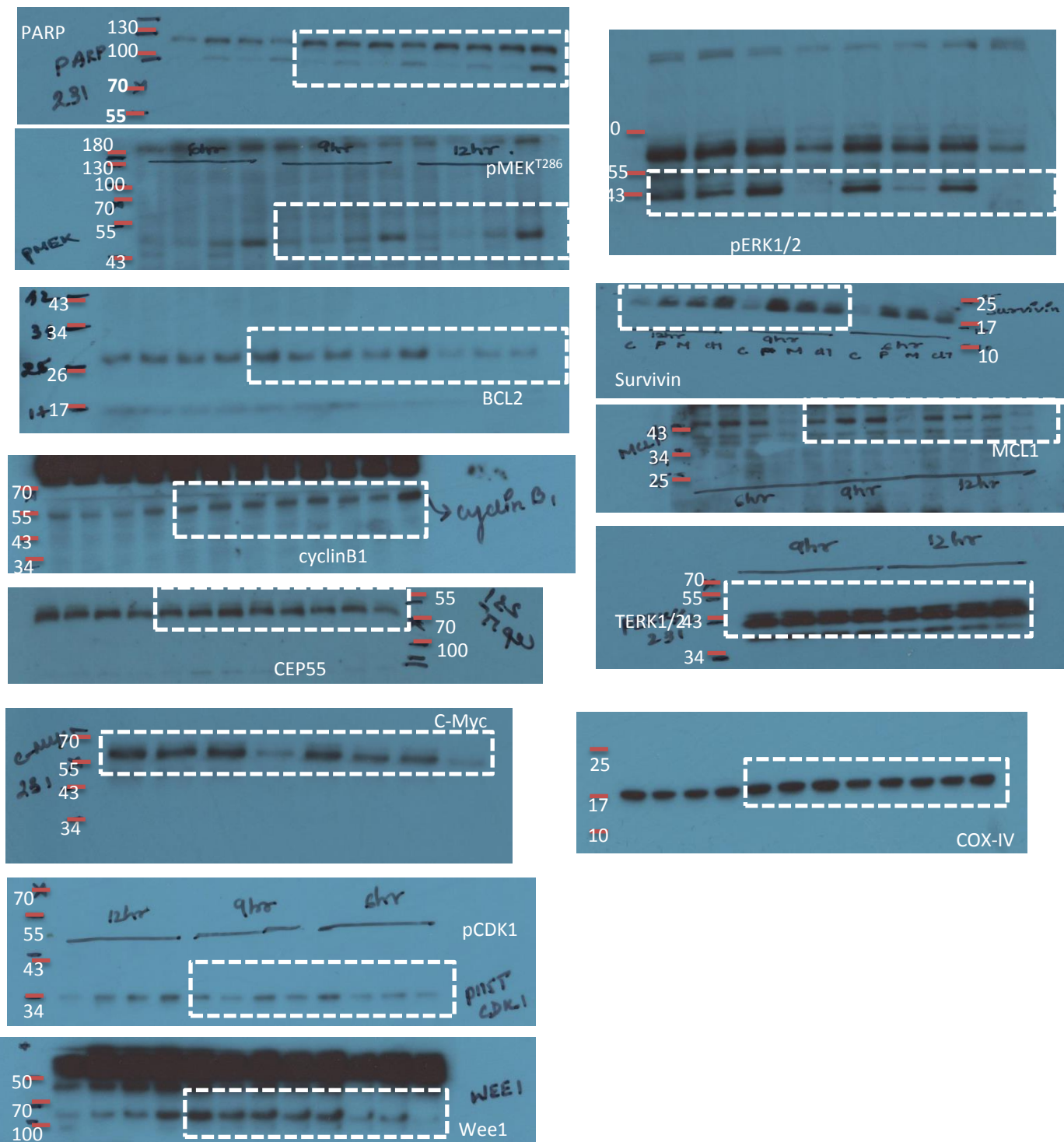

Figure 6D

SUM159PT

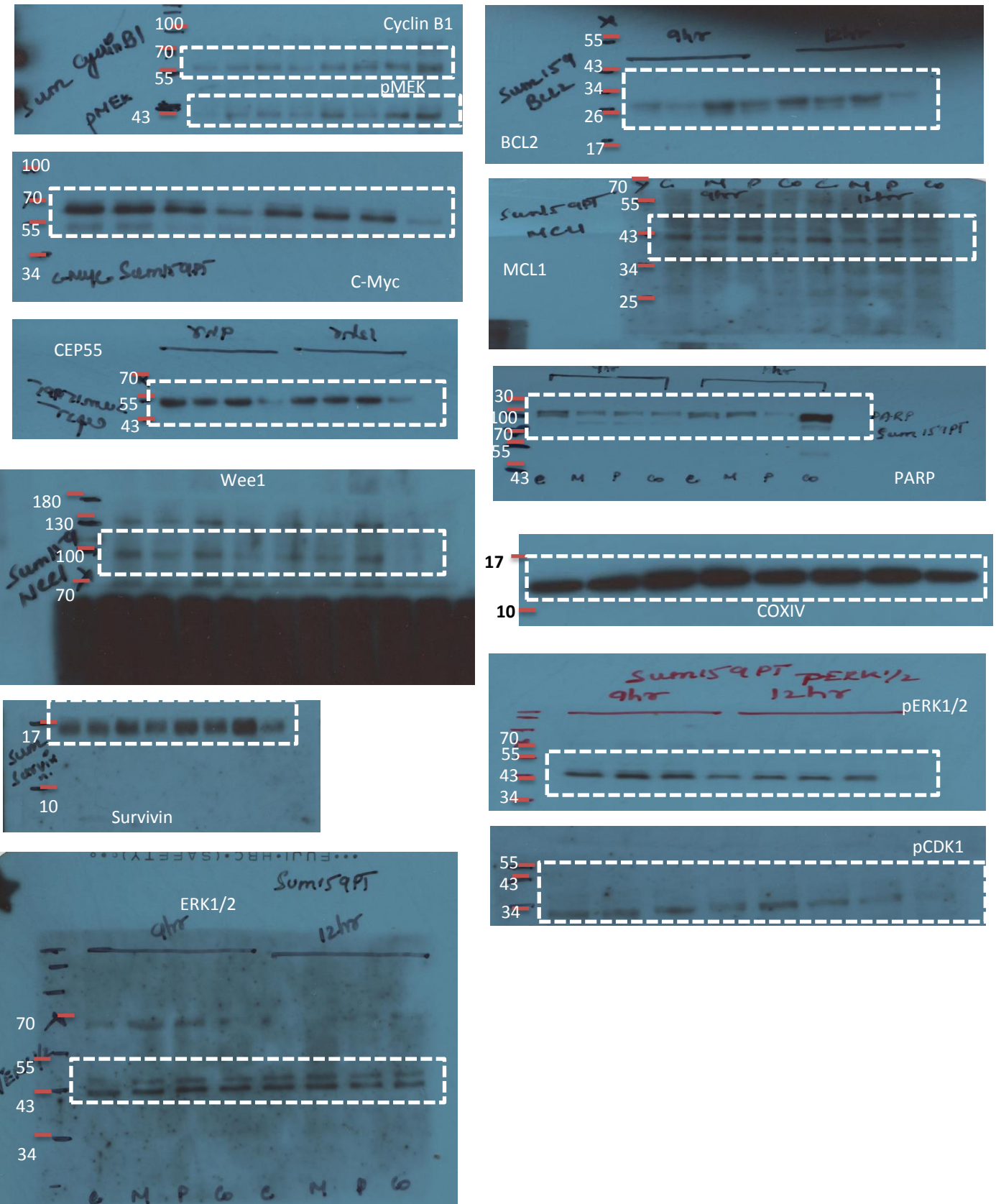

Supplement: Supplementary file 10 — Source Data for Figure 6 [file EMMM-10-e8566-s008.pdf]
